# The phage defence island of a multidrug resistant plasmid uses both BREX and type IV restriction for complementary protection from viruses

**DOI:** 10.1093/nar/gkab906

**Published:** 2021-10-18

**Authors:** David M Picton, Yvette A Luyten, Richard D Morgan, Andrew Nelson, Darren L Smith, David T F Dryden, Jay C D Hinton, Tim R Blower

**Affiliations:** Department of Biosciences, Durham University, Stockton Road, Durham DH1 3LE, UK; New England Biolabs, 240 County Road, Ipswich, MA 01938, USA; New England Biolabs, 240 County Road, Ipswich, MA 01938, USA; Department of Applied Sciences, University of Northumbria, Newcastle Upon Tyne NE1 8ST, UK; Department of Applied Sciences, University of Northumbria, Newcastle Upon Tyne NE1 8ST, UK; Department of Biosciences, Durham University, Stockton Road, Durham DH1 3LE, UK; Institute of Infection, Veterinary and Ecological Sciences, University of Liverpool, Liverpool L69 7ZB, UK; Department of Biosciences, Durham University, Stockton Road, Durham DH1 3LE, UK

## Abstract

Bacteria have evolved a multitude of systems to prevent invasion by bacteriophages and other mobile genetic elements. Comparative genomics suggests that genes encoding bacterial defence mechanisms are often clustered in ‘defence islands’, providing a concerted level of protection against a wider range of attackers. However, there is a comparative paucity of information on functional interplay between multiple defence systems. Here, we have functionally characterised a defence island from a multidrug resistant plasmid of the emerging pathogen *Escherichia fergusonii*. Using a suite of thirty environmentally-isolated coliphages, we demonstrate multi-layered and robust phage protection provided by a plasmid-encoded defence island that expresses both a type I BREX system and the novel GmrSD-family type IV DNA modification-dependent restriction enzyme, BrxU. We present the structure of BrxU to 2.12 Å, the first structure of the GmrSD family of enzymes, and show that BrxU can utilise all common nucleotides and a wide selection of metals to cleave a range of modified DNAs. Additionally, BrxU undergoes a multi-step reaction cycle instigated by an unexpected ATP-dependent shift from an intertwined dimer to monomers. This direct evidence that bacterial defence islands can mediate complementary layers of phage protection enhances our understanding of the ever-expanding nature of phage-bacterial interactions.

## INTRODUCTION

Bacteria are outnumbered 10-fold by the estimated ≥10^30^ phages on Earth (1,2), and are infected at a rate of 10^25^ per second (3). This vast predator–prey interaction has driven the evolution of varied means of protection (4–6). Examples include the well-established restriction-modification (R-M) (7), abortive infection (8) and CRISPR-Cas (9) systems. R-M systems have previously been observed clustered in ‘immigration control regions’ (10), and functional studies examined the differential responses from multiple R-M systems (11–13). Recent comparative genomic analyses have revealed that diverse phage-resistance genes are indeed commonly clustered into ‘defence islands’ (14,15). By coupling this clustering phenomenon with ‘guilt-by-association’ inference of function, many putative individual phage-resistance systems have been identified and characterised (16). This has in part contributed to the ongoing proliferation of studies focussing on individual systems including Bacteriophage Exclusion (BREX) (17), DISARM (18), BstA (19), prokaryotic Argonaute (20), bacterial cGAS (21), and SspABCD (22), amongst others. Further systematic studies are now needed that address the functional interplay between these diverse phage-resistance systems within defence islands.

One common pairing in identified defence islands (14,15) is between genes encoding Phage Growth Limitation (Pgl) systems (23) and GmrS/GmrD type IV restriction enzymes (24). Using the Pgl alkaline phosphatase gene, *pglZ*, to locate phage-resistance genes, the distinct BREX systems were identified in 10% of bacterial and archaeal genomes (17). BREX methyltransferases hemi-methylate non-palindromic 6 bp sequences on the N6 adenine nitrogen (N6mA) at the fifth position of the motif (17,25,26). This methylation marks host DNA, leaving incoming non-methylated DNA susceptible to BREX attack. The mechanistic basis of the prevention of phage proliferation by BREX is unknown. Whilst BREX targets non-modified DNA, type IV restriction enzymes such as GmrSD recognise and cleave modified DNA such as that from phage T4 (27). GmrS contains Domain of Unknown Function (DUF) 262, which is proposed to be involved in nucleotide hydrolysis (28). GmrD contains DUF1524, which is proposed to be an HNH nuclease domain (28). Whilst GmrSD systems were originally identified as being encoded by separate *gmrS* and *gmrD* genes, the predominant form is a single polypeptide produced from a *gmrSD* gene fusion (28–30). These GmrSD fused polypeptides can also sometimes be extended with a diverse array of additional DUF domains (28).

Type IV restriction enzymes include multiple unrelated families, with varied architectures and mechanisms, which provide specificity towards diverse DNA modifications (31). This includes 5-methyl cytosine (5mC) and 5-hydroxymethyl cytosine (5hmC) that have been recently identified in a widening range of eukaryotic systems, including mammalian stem cells and nerve tissues (32–34). Type IV enzymes are therefore being used to map epigenetic modifications, by allowing cleavage of targeted DNAs and analysis of the resulting fragments by next generation sequencing (35–37).

We have identified a phage defence island containing BREX and a type IV restriction enzyme, encoded on a multidrug resistant plasmid of the emerging animal and human pathogen *Escherichia fergusonii* (38). The interplay between individual phage-resistance systems encoded together within a phage defence island is not currently well understood. Using a suite of 30 coliphages isolated during undergraduate practical classes, we discovered that an atypically large BREX operon offers complementary protection against a broad range of phages when associated with the type IV GmrSD homologue, BrxU. Subsequent analyses of BrxU have produced the first structures of the GmrSD family, and provided biochemical insight into a complex reaction cycle of DNA-modification dependent cleavage.

## MATERIALS AND METHODS

### Bacterial strains and culture conditions

**Figure 1.**
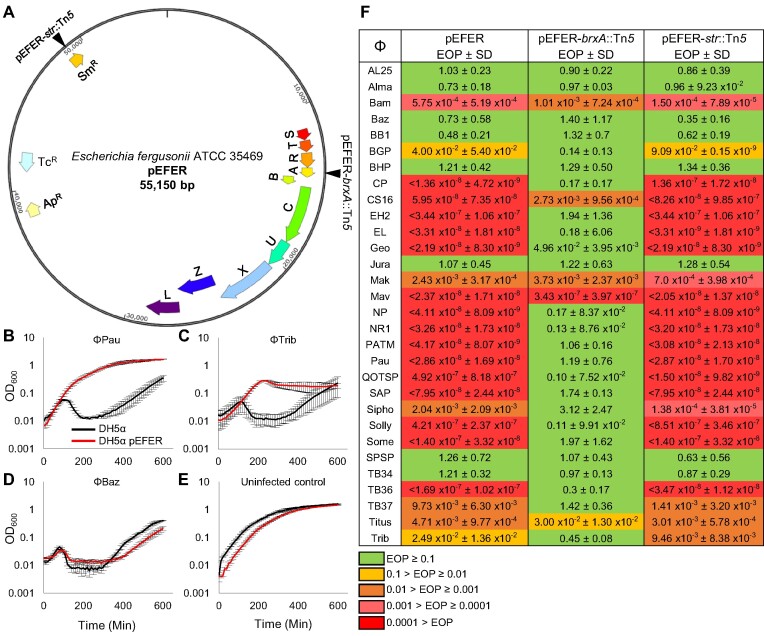
Plasmid pEFER confers effective phage defence. (**A**) Plasmid map of *Escherichia fergusonii* ATCC 35469 pEFER, showing putative defence island and antibiotic resistance genes. Transposon insertion sites are indicated for pEFER-*brxA*::Tn*5* and pEFER-*str*::Tn*5*. BrxB and PglZ are in a different reading frame from other genes in the defence island. (**B**–**E**), Growth curves of *E. coli* DH5α (black) and *E. coli* DH5α pEFER (red), infected with phages or an uninfected control. Bacterial cultures were grown to mid exponential phase and diluted to a starting OD_600_ of 0.05. Cultures were inoculated at an MOI of 0.001 at time point 0 min and absorbance was measured every 10 min. Experiments were run in triplicate and the mean values are plotted. Error bars represent the standard deviation of replicates. (**F**) Efficiency Of Plating (EOP) values for *E. coli* DH5α strains containing pEFER or transposon mutants of pEFER, pEFER-*brxA*::Tn*5* and pEFER-*str*::Tn*5*, against a suite of environmentally isolated coliphages. The control strain was *E. coli* DH5α WT. Values are mean EOPs from triplicate data, shown with standard deviation. Values presented with < extended below the range of this assay and no plaques were observed.

Total genomic DNA (gDNA) of *E. fergusonii* ATCC 35469 was obtained from ATCC. *Escherichia coli* strains DH5α (ThermoFisher Scientific), BL21 (DE3) (ThermoFisher Scientific), NEB2796 (New England Biolabs, NEB) and ER2796 (39) were grown at 37°C, either on agar plates or shaking at 220 rpm for liquid cultures. Luria broth (LB) was used as the standard growth media for liquid cultures, and was supplemented with 0.35% (w/v) or 1.5% (w/v) agar for semi-solid and solid agar plates, respectively. Growth was monitored using a spectrophotometer (WPA Biowave C08000) measuring optical density at 600 nm (OD_600_). When necessary, growth media was supplemental with ampicillin (Ap, 50 μg/ml), kanamycin (Km, 50 μg/ml), streptomycin (Sm, 100 μg/ml), tetracycline (Tc, 10 μg/ml), isopropyl-β-d-thiogalactopyranoside (IPTG, 1 mM), l-arabinose (l-ara, 0.1% w/v) or d-glucose (glu, 0.2% w/v). For growth curves (Figure 1), single colonies were used to inoculate overnight cultures of LB incubated at 37°C. The overnight culture was used to inoculate 200 μl of LB at an OD_600_ of 0.01 in the corresponding well of a sterile 96-well plate. Cultures were infected with phage stocks to a Multiplicity of Infection MOI of 0.001. Plates were incubated at 37°C within a SPECTROstar Nano plate reader (BMG Labtech) shaking at 400 rpm. OD_600_ was measured at 10 min intervals and the mean average for each time point was calculated from three biological repeats. Data shown are mean values with standard deviation represented by error bars.

### Isolation and use of environmental coliphages

All *E. coli* bacteriophages were isolated from freshwater sources in Durham city centre and the surrounding areas. Water sampling and the first round of phage isolation was performed as an undergraduate laboratory practical session at Durham University. A 10 ml water sample was filtered through a 0.22 μm filter to remove debris. Filtrates were supplemented with 10 ml of LB, and then inoculated with 1 ml of an overnight culture of *E. coli* DH5α. Cultures were grown for 3 days before a 1 ml aliquot was transferred to a sterile microcentrifuge tube and centrifuged at 12 000 × g for 5 min at 4°C. The supernatant was transferred to a new microcentrifuge tube and 100 μl of chloroform was added to kill any remaining bacteria. Samples were vortexed before a serial dilution series was prepared in phage buffer (10 mM Tris–HCl pH 7.4, 10 mM MgSO_4_, 0.1% gelatin). 10 μl of serial dilutions and 200 μl of *E. coli* DH5α were added to 4 ml of semi-solid LB agar in a sterile 7 ml plastic bijou. Samples were gently mixed and poured on to LB agar plates. Plates were incubated overnight and individual plaques were harvested the following morning using sterile toothpicks into 200 μl phage buffer. Serial dilutions were re-prepared and a series of agar plates were poured as detailed above. These steps were repeated as necessary to ensure a single plaque morphology was observed for all dilutions. Lysates were then prepared by scraping a confluent lawn of phage plaques into a 50 ml centrifuge tube. 3 ml of phage buffer and 500 μl of chloroform was added before mixing with a vortex for 2 min. Samples were incubated at 4°C for 30 min before being centrifuged at 4000 x g for 20 min. The supernatant was transferred to a sterile glass bijou and 100 μl of chloroform was added. Phage lysates were stored at 4°C. Efficiency Of Plating (EOP) values were calculated by determining the phage titre on a test strain, divided by the titre on a control strain. The EOP values obtained used *E. coli* DH5α WT as control strain throughout, to allow direct comparisons between the multiple plasmid backbones used in test strains. EOP data were collected in triplicate and the mean value is shown along with standard deviation.

### DNA isolation and manipulation

Analysis of pEFER was performed using BLAST (40). Further amino acid sequence alignments were performed using PROMALS3D (41). All oligonucleotide primers used in this study (Supplementary Table S3) were obtained from Integrated DNA Technologies. PCR amplicons and plasmids were purified using Monarch DNA kits (NEB). PCR, restriction digests, ligations, transformations and agarose gel electrophoresis were performed using standard molecular biology techniques. Constructed plasmids were confirmed via sequencing with an Abi 3370 DNA sequencer. The pSAT1-LIC-*brxU*^+^ expression construct adds a cleavable N-terminal His_6_-SUMO tag, and was generated via Ligation Independent Cloning (LIC). Primers TRB851 and TRB852 were used to amplify *brxU* from pEFER for insertion into pSAT1-LIC (42) to produce pTRB446. Primers TRB865 and TRB866 were used to amplify *brxU* from pEFER which was inserted into pBAD30 to produce pBAD30-*his_6_-brxU*^+^. Golden Gate Assembly (GGA) (43) was performed to produce pBrxXL and its derivatives. QuikChange mutagenesis was performed to remove BsaI sites from pUC19 (NEB) to produce pTRB479. The pEFER BREX locus was partitioned into multiple ∼3–4 kb regions and amplified using primers with flanking BsaI sites with unique overhang sequences (Supplementary Table S3). pTRB479 and inserts were digested with BsaI and ligated with T4 DNA ligase to create GGA donor constructs. A 3:1 insert: vector ratio was used in a GGA reaction containing BsaI and T4 DNA ligase. Positive colonies of transformed *E. coli* DH5α were selected for on LB agar containing ampicillin. Transposon mutagenesis was performed using EZ::Tn*5*^TM^ < Kan-2 > according to the manufacturer's instructions (Cambridge Bioscience). Insertion events were mapped using random-primed PCR (44) and DNA sequencing. Site directed mutants of pBAD30-*his_6_-brxU*^+^ were designed and GenScript were commissioned to generate the constructs. To produce synthetically modified substrates for BrxU (Figure 3B and Supplementary Figure S3), PCR was performed using primers TRB1434 and TRB1435 to produce a 2.7 kb amplicon with pUC19 as a template, replacing dCTP when needed with either 5mC or 5hmC (Jena Biosciences) to produce DNA substrates containing the desired modification. Amplicons were analysed by agarose gel electrophoresis and purified using a Monarch DNA gel extraction kit (NEB). DNA containing 5hmC was subsequently treated with either β-glucosyltransferase (NEB), using UDP-glucose as a donor, to produce glc-5hmC DNA, or with T4 Polynucleotide Kinase (NEB) and T4 DNA Ligase (NEB), to produce a circularised form, prior to re-purification. Phage gDNA was isolated via phenol-chloroform extraction and purified using ethanol precipitation. Bacterial gDNA for PacBio sequencing was isolated using the Quick-DNA Miniprep Plus Kit (Zymo Research).

### Pacific biosciences sequencing

Libraries for sequencing were prepared using the SMRTbell Template Prep kit 1.0 (Pacific Biosciences). Briefly, purified bacterial gDNA was sheared using gTubes (Covaris) to produce DNA fragments with a mean size of 5–10 kb. The DNA was damage repaired, end repaired and ligated to SMRT-bell adapters. Non SMRT-bell DNAs were removed by exonuclease treatment. Sequencing was performed on either a PacBio RSII or a Sequel I sequencer (Pacific Biosciences). Data were analysed using PacBio SMRTAnalysis 2.3.0 Modification_and_Motif_Analysis_1.0 for RSII data, or SMRTLink_6.0 software Base Modification Analysis for Sequel data, to identify DNA modifications and their corresponding target motifs.

### Protein expression and purification

For expression of target proteins, *E. coli* BL21 (DE3) was transformed with pSAT1-LIC or pBAD30 expression constructs. A single colony was used to inoculate 25 ml LB containing Ap and grown overnight at 37°C. Overnight cultures were used to inoculate six 2 l baffled flasks containing 1 l 2× YT supplemented with Ap. Cultures were grown at 37°C shaking at 180 rpm until an OD_600_ of ∼0.6 at which point protein expression was induced. For pSAT1-LIC-*brxU*^+^, expression was induced by the addition of IPTG to a final concentration of 1 mM and cultures were grown overnight at 18°C. For pBAD30-*his_6_-brxU*^+^, expression was induced by the addition of l-ara to a final concentration of 0.1% (w/v) and grown for 4 h at 37°C. Selenomethionine (SM)-labelled protein expression was performed using the SM Medium expression kit (Molecular Dimensions). pSAT1-LIC-*brxU*^+^ was used to transform *E. coli* BL21 (DE3) and a single colony was used to inoculate a 5 ml SM Complete Medium starter culture containing Ap, which was grown for 36 h at 37°C. Starter cultures were used to inoculate 1 l of SM Complete Medium in a 2 l baffled flask to an OD_600_ of 0.05 and cultures were grown at 37°C until an OD_600_ of 0.6–0.8, at which point 10 ml of 100× methionine inhibitory feedback mix (10 g/l lysine, phenylalanine, threonine and 5 g/l leucine, isoleucine, valine and L-SM) was added. 30 min after the addition of inhibitory feedback mix, IPTG was added to a final concentration of 1 mM to induce protein expression. Cultures were grown overnight at 18°C. Cultures were pelleted at 5000 × g for 30 min at 4°C. Cell pellets were resuspended in 50 ml of ice-cold A500 (20 mM Tris–HCl pH 7.9, 500 mM NaCl, 10 mM imidazole and 10% glycerol) and used immediately or flash frozen in liquid nitrogen and stored at −80°C.

Pellets were lysed via sonication and centrifuged at 45 000 × g at 4°C for 30 min. All clarified cell lysates were passed over a 5 ml HisTrap HP column (Cytiva), and washed with 50 ml of A500. His_6_-BrxU WT and mutants expressed from pBAD30-*his_6_-brxU*^+^ were further washed with 50 ml of W500 (20 mM Tris–HCl pH 7.9, 500 mM NaCl, 40 mM imidazole and 10% glycerol) and eluted from the column in B500 (20 mM Tris–HCl pH 7.9, 500 mM NaCl, 250 mM imidazole and 10% glycerol). Imidazole was removed via dialysis and samples were stored in glycerol (30% w/v) at −80°C. His_6_-SUMO-BrxU expressed from pSAT1-LIC-*brxU*^+^ was treated with hSENP2 SUMO protease and dialysed overnight into A100 (20 mM Tris–HCl pH 7.9, 500 mM NaCl, 10 mM imidazole and 10% glycerol). The resulting untagged BrxU was loaded on to a second 5 ml HisTrap HP column and the flowthrough was collected and loaded on to a 5 ml HiTrap Q HP column (Cytiva). The Q HP column was transferred to an Äkta Pure (Cytiva) FPLC system and BrxU was eluted from the column over a gradient from 100% A100 to 100% C1000 (20 mM Tris–HCl pH 7.9, 1000 mM NaCl, 10 mM imidazole and 10% glycerol). Fractions were analysed via SDS-PAGE and peak fractions were pooled before being resolved via size exclusion through a Sephacryl S-300 HR gel filtration column in preparative SEC buffer (20 mM Tris–HCl pH 7.9, 500 mM KCl and 10% glycerol). Fractions were analysed via SDS-PAGE and peak fractions were pooled. The pooled sample was dialysed into Xtal buffer (20 mM Tris–HCl pH 7.9, 200 mM NaCl and 2.5 mM DTT). Untagged BrxU for crystallisation was used immediately, untagged BrxU samples for biochemical analysis were stored in glycerol (30%, w/v) at −80°C.

### DNA cleavage assays

2 μl of 100 ng/μl gDNA was added to a mixture of 2 μl 10× DPMG buffer (200 mM Tris–HCl pH 7.5, 500 mM CH_3_COOK, 100 mM MgSO_4_), 2 μl 10 mM ATP, 5 μl of 2 μM BrxU and 9 μl of nuclease free water. Nuclease free water was used for negative controls in place of ATP. For metal-dependency assays (Figure 3C), Mg^2+^ was excluded from the sample buffer and replaced with the relevant cation. For nucleotide-dependency assays (Figure 3D, E), the nucleotide was substituted as required. Samples were incubated at 37°C for 30 min and reactions were terminated by incubating at 75°C for 10 min. Agarose gels were resolved at 120 V for 45 min in 1× TAE buffer containing 0.5 μg/ml EtBr. Gels were visualised with BioRad Image Lab software. All experiments were run in triplicate with replicates on individual gels, with a single gel shown for each experiment as representative of replicates.

### Nucleotide hydrolysis assays

Nucleotide hydrolysis was determined by measuring the concentration of released P_i_, and assays were performed according to the BIOMOL green (Enzo Life Sciences) protocol. A 96-well plate format was used and columns 1 and 2 were used for phosphate standard serial dilutions to allow for calculation of experimental results. Experimental wells were set up in 50 μl total volumes. Each well contained 5 μl 10× DPMG buffer, 5 μl of 1 mM NTP and 5 μl of 5 μM protein, made up to 50 μl with MilliQ (MQ) water. 5 μl was added for each additional reagent (EDTA, DNA) in place of equal volumes of MQ water. EDTA was used at a final concentrations of 10 mM, and 100 ng of respective substrate DNA was used. All experiments were incubated at 37°C for 30 min shaking at 400 rpm and reactions were terminated by the addition of 100 μl of BIOMOL green reagent (Cambridge Bioscience) and immediately transferred to a SPECTROstar plate reader. Plates were incubated at 30°C shaking at 400 rpm whilst the BIOMOL green reagent developed. Absorbance at 620 nm was measured after 30 min. Data shown are mean values averaged from three technical replicates with error bars representing standard deviation.

### Analytical gel filtration

A Superdex 200 Increase GL 5/150 (Cytiva) was connected to an ÄKTA Pure system (Cytiva) and equilibrated by running through 2 column volumes of filtered MQ water and 5 column volumes of analytical SEC buffer (20 mM Tris–HCl pH 7.9 and 150 mM NaCl) at 0.175 ml/min. It was then calibrated using standard calibration kits (Cytiva). The resulting calibration curve was used to determine dimer and monomer states of BrxU according to elution volume and molecular weight (67.9 kDa for a BrxU monomer). A 50 μl sample was prepared containing 5 μl of 5 μM BrxU and 5 μl of 10× DPMG buffer, and made up to 50 μl with MQ water. For samples without MgSO_4_, DPMG buffer was replaced by DPMG-buffer (200 mM Tris–HCl pH 7.5, 500 mM CH_3_COOK). 5 μl of 10 mM nucleotide was used to replace 5 μl of MQ water in nucleotide-containing samples (Figure 3G). For analysis of BrxU mutants, all samples were prepared containing 5 μl protein, 5 μl 10× DPMG buffer and either 5 μl of 10 mM ATP or 5 μl of MQ water, and made up to a total volume of 50 μl with MQ water (Figure 5D). These 50 μl samples were incubated at 37°C for 15 min and then loaded to overfill a 10 μl loop via a 50 μl Hamilton syringe. Two column volumes of analytical sizing buffer was run through the sample loop directly on to the column at 0.175 ml/min. Absorbance at 280 nm was measured corresponding to the eluting protein signal. Experiments were performed a total of three times and a single dataset is shown as representative of the triplicate data.

### Protein crystallisation

Native and SM-derivatised BrxU were concentrated to a final 12 mg/ml in Xtal buffer with the addition of 5 mM MgSO_4_ and 2 mM AMP-PnP, and crystallisation trials were performed with commercially available screens (Molecular Dimensions). Using a Mosquito Xtal3 robot (SPT Labtech), drops were set at 200:100 nl and 100:100 nl (protein : precipitant) ratios at 18°C. Crystals were visible after 24 hours in Pact Premier G8 (0.2 M Na_2_SO_4_, 0.1 M Bis–Tris propane pH 7.5 and 20% w/v PEG 3350). Optimised crystal formation was observed when substituting 0.2 M Na_2_SO_4_ for 0.2 M (NH_4_)_2_SO_4_, resulting in crystals from which both native and SM-derivatised data were obtained. Crystals were harvested by mounting into nylon cryo-loops and soaking in a drop of 2:1 ratio of reservoir liquid and cryo solution (20 mM Tris–HCl pH 7.9, 150 mM NaCl, 2.5 mM DTT and 80% glycerol), then flash freezing in liquid nitrogen.

### X-ray data collection and structure determination

Diffraction data were collected at Diamond Light Source (DLS) using beamlines I24 (native 2.12 Å and SM-derivatised 2.70 Å data) and I04 (native 2.85 Å data) (Supplementary Table S2). Two individual 360° datasets were collected from a single native crystal and merged using the DIALS pipeline in iSpyB (Diamond Light Source) to obtain data at 2.12 Å. An additional 5 datasets were obtained from a different, individual crystal and merged to produce another native dataset at 2.85 Å. A total of 15 datasets were collected from 4 SM-derivatised BrxU crystals at the selenium peak (0.9786 Å) and merged to produce a dataset at 2.70 Å. Merged data were processed via XDS (45) and spacegroups were corroborated using AIMLESS from CCP4 (46). The crystal structure of SM-BrxU was solved via SAD using the SHELX (47) suite in CCP4 (46). The starting model was then built using BUCCANEER (48) and REFMAC (49). The resulting model was then used as a search model for molecular replacement by PHASER (50), to solve both the native 2.12 and 2.85 Å structures. The initial outputs from molecular replacement were again built in BUCCANEER (48), then iteratively refined and re-built using PHENIX (51) and COOT (52), respectively. The quality of the final model was assessed using PHENIX, COOT and the wwPDB validation server. Structural figures were generated using PyMol (Schrödinger).

## RESULTS

### Discovery of the pEFER phage defence island

The initial study into BREX used comparative genomics to compile a list of type I BREX systems (17). We selected the plasmid-borne type I BREX system from *E. fergusonii* ATCC 35469 as a suitable candidate to investigate phage defence islands (Figure 1A). The 55.15 kb pEFER plasmid contains 58 ORFs, including an extended BREX locus, multiple antibiotic-resistance genes, a toxin-antitoxin system, a partitioning system for plasmid replication, and a varied set of transposases (Figure 1A and Supplementary Table S1). The putative ∼18 kb BREX locus contains the canonical *brxA, brxB, brxC, pglX, pglZ* and *brxL* genes, with three additional upstream genes we have named *brxR, brxS* and *brxT* (Figure 1A). BrxR is a WYL-domain predicted transcriptional regulator, BrxS is a putative IS3 family transposase and BrxT is a hypothetical protein of unknown function. A further gene, *brxU*, not part of the canonical BREX system (17), is inserted between *brxC* and *pglX*. The BrxU protein contains DUF262 and DUF1524 domains, and shares 13% amino acid sequence identity with the single polypeptide type IV restriction enzyme GmrSD (30) (Supplementary Figure S1). The presence of the putative type IV restriction enzyme BrxU within the BREX locus raised the possibility that pEFER encodes a novel multifunctional phage defence island.

To test for activity against phages, *E. coli* DH5α was transformed with a total genomic DNA (gDNA) extraction from *E. fergusonii* ATCC 35469, taking advantage of the pEFER-encoded tetracycline resistance for plasmid selection. *E. coli* DH5α pEFER and *E. coli* DH5α wild type (WT) were then grown in the presence of a range of environmentally-isolated coliphages (Figure 1B–D). Plasmid pEFER provided resistance against phage φPau and φTrib (Figure 1B/C), but not phage φBaz (Figure 1D). The uninfected controls reached the same final OD_600_, though *E. coli* DH5α pEFER lagged slightly behind WT (Figure 1E).

Phages φPau, φTrib and φBaz were part of a larger suite of thirty environmental coliphages that were isolated from the River Wear and other waterways around Durham, UK, by undergraduates completing a final year microbiology practical module in 2016 and 2017 (see Acknowledgements). To quantify the level of phage defence, the Efficiency Of Plating (EOP) of all thirty phages was determined against *E. coli* DH5α pEFER, using *E. coli* DH5α WT as the control (Figure 1F). EOP is the relative number of plaques that a phage stock is capable of producing on a particular bacterial strain (53). Plasmid pEFER reduced the EOP for 22 of the 30 phages (Figure 1F). The scale of effect varied, with the EOP for phages such as φCP and φPau reduced by > eight orders of magnitude on *E. coli* DH5α pEFER (resulting in zero plaques), whereas phages such as φBGP and φTrib only had a 100-fold reduction (Figure 1F). As expected, φBaz did not exhibit a reduced EOP (Figure 1D/F).

To confirm that phage defence was defence island-dependent, *in vitro* transposon mutagenesis was performed on plasmid pEFER. The resulting transformants were screened for a loss of phage defence using φPau. Transformants were also screened for streptomycin susceptibility, to identify a second, isogenic, plasmid for comparison. The identified mutants were sequenced by random-primed PCR to map the insertion sites (44). Clone pEFER-*brxA*::Tn*5* has a transposon insertion within *brxA*, and clone pEFER-*str*::Tn*5* carries a transposon insertion within *strB* (Figure 1A). The pEFER-*brxA*::Tn*5* and pEFER-*str*::Tn*5* plasmids were then tested for phage defence using the same suite of phages (Figure 1F). The pEFER-*brxA*::Tn*5* insertion ablated phage defence activity against 17 of the 22 previously susceptible phages, confirming that the putative defence island did indeed provide phage defence (Figure 1F). As five phages, φBam, φCS16, φMak, φMav and φTitus, still had a reduced EOP with pEFER-*brxA*::Tn*5*, we inferred either that the transposon insertion did not fully remove activity from BREX and BrxU, or another phage-resistance system could be encoded elsewhere on pEFER. Interestingly, the EOP of φCS16 changed from an eight-log reduction on *E. coli* DH5α pEFER, to a three-log reduction on *E. coli* DH5α pEFER-*brxA*::Tn*5*, raising the possibility that multiple systems were targeting φCS16 (Figure 1F). The isogenic control plasmid pEFER-*str*::Tn*5* conferred the same EOP profile as progenitor plasmid pEFER (Figure 1F). Taken together, our data show that the BREX/BrxU phage defence island of pEFER provides a highly effective level of defence against a broad range of phages.

### pEFER phage defence is mediated by both BREX and BrxU

**Figure 2.**
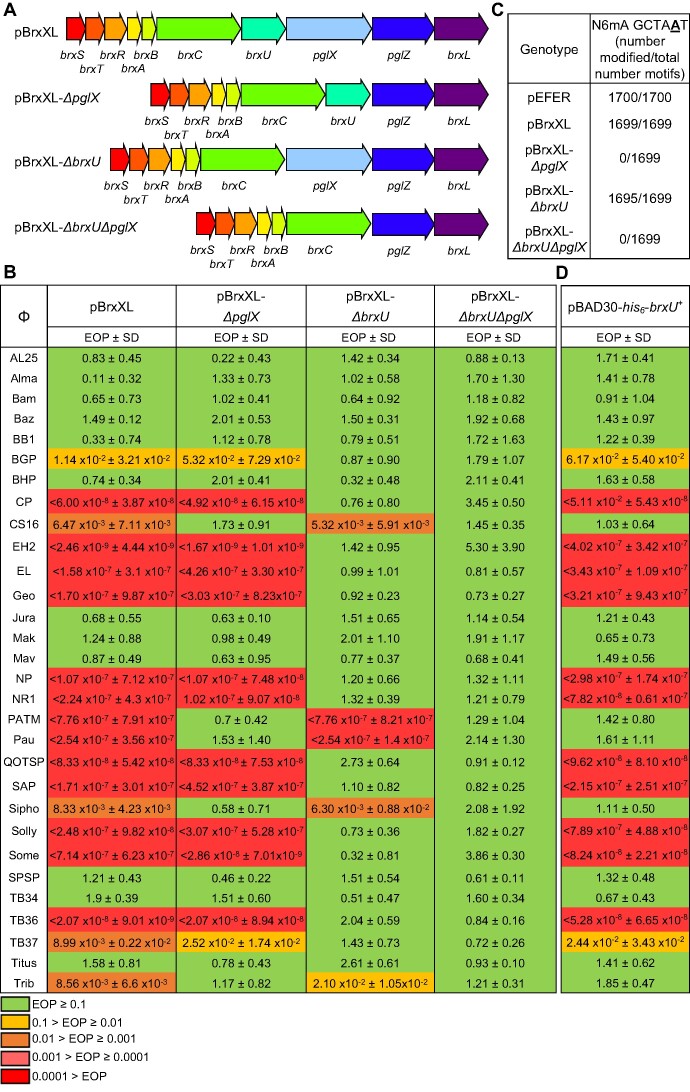
pEFER phage defence island encodes active BREX and BrxU systems. (**A**) Linear representation of phage defence island and subsequent mutant constructs. (**B**) EOP values for phage defence island constructs in (A). (**C**) PacBio methylome sequencing outputs for *E. coli* DH5α pEFER and *E. coli* ER2796 strains containing pBrxXL or mutant derivatives. (**D**) EOP values for pBAD30-*his_6_*-*brxU*^+^. Expression of BrxU was induced by the addition of 0.1% l-arabinose. Values are mean EOPs from triplicate data, shown with standard deviation. Values presented with < extended below the range of this assay and no plaques were observed.

To characterise the specific phage defence mechanisms, the entire ∼18 kb pEFER phage defence island was sub-cloned in sections, and then inserted into plasmid pGGA by Golden Gate Assembly (GGA) (43), yielding plasmid pBrxXL (Figure 2A). Plasmid pBrxXL provided the reciprocal activity of pEFER-*brxA*::Tn*5* (Figures 1F and 2B), reducing the EOP of 17 out of 30 phages to the same level as plasmid pEFER, and also modulating the EOP of φCS16. Because pBrxXL encodes the defence island, we hypothesised that the phage defence phenotype was dependent on both a BREX mechanism and the putative type IV restriction enzyme BrxU. The function of BREX relies upon the activity of the methyltransferase PglX, and so plasmids were made that contained either a deletion of *pglX*, *brxU*, or the double mutant *ΔbrxUΔpglX* (Figure 2A). The mutant plasmids were then tested against the suite of coliphages, which demonstrated that the phages fall into two classes, one that was targeted by BrxU, and therefore had reduced EOP only on pBrxXL-*ΔpglX*, and another that was targeted by BREX and had a reduced EOP only on pBrxXL-*ΔbrxU* (Figure 2B). The double pBrxXL-*ΔbrxUΔpglX* mutant did not provide any protection against phage attack, supporting our hypothesis that this defence island carries two phage resistance mechanisms: the canonical PglX-mediated BREX system, and a novel mechanism mediated by BrxU (Figure 2B). Furthermore, as pBrxXL-*ΔbrxU* reduced the EOP of φCS16 by three orders of magnitude, compared to the eight orders of magnitude of protection provided by pEFER (Figure 1F), and pEFER reduced the EOP of four phages not targeted by pBrxXL (φBam, φMak, φMav and φTitus) (Figure 1F), we suspect that pEFER also encodes a third phage defence system outside the BREX/BrxU phage defence island, which awaits future characterisation.

PglX methyltransferases produce hemi-methylated DNA by generating N6-methylated adenines (N6mA) at the fifth position within 6-bp non-palindromic sequences of host DNA (17). The *Bacillus cereus* PglX targets TAGGAG (17) and the *E. coli* equivalent, BrxX, targets GGTAAG (25). Pacific Biosciences (PacBio) sequencing of gDNA from *E. fergusonii* ATCC 35469 identified N6mA modifications at the fifth adenine of 1810/1812 (99.89%) of genome-wide GCTAAT motifs, alongside modifications from Dam (54) and four type I R-M systems (55). The GCTAAT modification was then observed in gDNA extracts from *E. coli* DH5α pEFER (Figure 2C), along with Dam and EcoKI (56) methylation sites. To eliminate all *E. coli* methylation from the analyses, subsequent PacBio sequencing was performed using the methylation-deficient *E. coli* strain ER2796 (39). This analysis identified a single modification motif, GCTAAT N6mA, in *E. coli* ER2796 strains containing pBrxXL or pBrxXL-*ΔbrxU*, but not pBrxXL-*ΔpglX* or pBrxXL-*ΔbrxUΔpglX* (Figure 2C). Our findings show that the pEFER-encoded phage defence island encodes a PglX-dependent BREX system.

Surprisingly, Figure 2B showed that of the 18 phages with reduced EOPs, only five were BREX-sensitive; φCS16, φPATM, φPau, φSipho and φTrib (though the scale of impact remained an impressive seven orders of magnitude in the cases of φPATM and φPau). As the remaining 13 phages with reduced EOPs appeared BrxU-sensitive (Figure 2B), we chose to further characterise BrxU. First, to confirm that BrxU alone is sufficient to provide phage defence, we inserted a hexahistidine-tagged WT *brxU* gene (*his_6_*-*brxU*) into a pBAD vector and once again tested it against our suite of coliphages (Figure 2D). The EOP profile on *E. coli* DH5α pBAD30-*his_6_*-*brxU*^+^ was the same as that on *E. coli* DH5α pBrxXL-*ΔpglX* (Figure 2B, D), demonstrating that BrxU was indeed sufficient for phage defence, and that the tag had no impact on activity. Thus our phage defence island carries two distinct phage resistance mechanisms, one PglX-dependent and one mediated by BrxU.

### BrxU is a promiscuous type IV restriction enzyme

**Figure 3.**
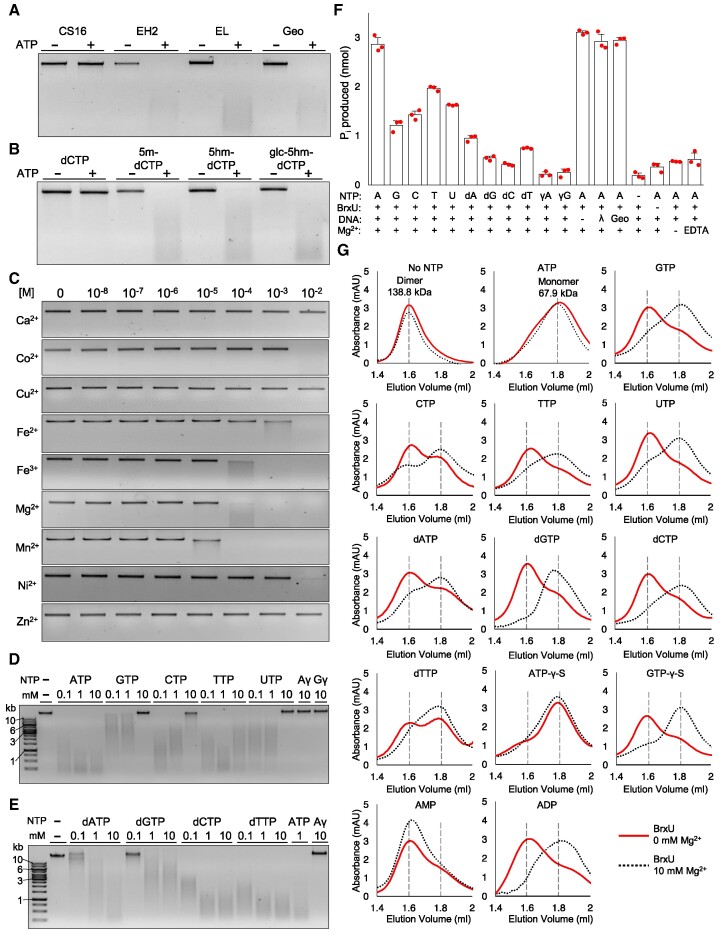
BrxU is a promiscuous type IV restriction enzyme. (**A**) Cleavage of phage φEH2, φEL and φGeo gDNA by untagged BrxU. The gDNAs of phages with reduced EOP values in the presence of pBAD30-*his_6_*-*brxU*^+^ (Figure 2D) were cleaved by BrxU *in vitro*. The gDNA of BrxU-insensitive phage φCS16 was not cleaved. All lanes contain 500 nM BrxU and 10 mM MgSO_4_, with and without 1 mM ATP. All phage gDNAs were tested in this manner (Supplementary Figure S2). (**B**) DNA cleavage assays show untagged BrxU degrades DNA containing 5mC, 5hmC and glc-5hmC. BrxU does not degrade DNA that does not incorporate modified cytosines. All lanes contain 500 nM BrxU and 10 mM MgSO_4_, with and without 1 mM ATP. (**C**) Metal-dependent cleavage of φGeo gDNA by untagged BrxU. Metal concentration is shown in M, as a titration with 500 nM BrxU and 1 mM ATP. (**D**-**E**) Nucleotide-dependent cleavage of φGeo gDNA by BrxU. All lanes contain 500 nM BrxU and 10 mM MgSO_4_. Nucleotide concentration is shown above each lane. Non-hydrolysable analogues ATP-γ-S (Aγ) and GTP-γ-S (Gγ) are shown as controls and cannot be utilised by BrxU for DNA cleavage. All gel data (A-E) are representative of triplicate experiments and samples were resolved in 1% agarose TAE at 120 V for 45 min. (**F**) Nucleotide hydrolysis assays with untagged BrxU. φBGP gDNA was the default DNA substrate. Presented data are the mean and standard deviations from triplicate experiments, with data points overlaid. (**G**) Analytical size exclusion analysis of untagged BrxU. 10 μl samples of 500 nM BrxU and nucleotides at 1 mM, with and without 10 mM MgSO_4_, were resolved at 0.175 ml/min. Traces in red represent samples that do not contain MgSO_4_. Dashed traces in black represent samples that were pre-incubated at 37°C with 10 mM MgSO_4_ prior to loading. Traces are representative of triplicate data, and relative elution positions for the dimeric and monomeric forms of BrxU are indicated by dashed grey lines. Untagged BrxU used in all panels was expressed from pSAT1-LIC-*brxU*^+^.

Single polypeptide type IV restriction enzymes recognise and degrade double-stranded DNAs containing modified cytosines (30). To determine whether BrxU was a restriction enzyme, we over-expressed, purified and incubated untagged BrxU protein with extracted phage gDNAs and magnesium, in the presence and absence of ATP (Figure 3A and Supplementary Figure S2). Genomic DNAs from the BrxU-sensitive phages (Figure 2D) were degraded by BrxU in an ATP-dependent manner (such as for φEH2, φEL and φGeo), whereas gDNAs from BrxU-insensitive phages (such as φCS16), were not degraded (Figure 3A and Supplementary Figure S2).

As GmrSD homologues have been shown to cleave DNAs containing modified cytosines (30), a range of modified 2.7 kb DNA substrates were generated using pUC19 as a template, to include either 5mC, 5hmC or glucosyl-5-hydroxymethyl cytosine (glc-5hmC) modifications. These were then tested against BrxU, which recognised and degraded DNA containing any of the three modifications in an ATP-dependent manner, but did not degrade unmodified DNA (Figure 3B). BrxU cleavage produced a smear of low molecular weight fragments, and no specific banding pattern (Figure 3B). BrxU degraded both linear and circular modified DNA, consistent with an endonuclease activity (Supplementary Figure S3).

As magnesium was required for the nucleolytic activity of BrxU (Figure 3A/B), nine divalent cations were titrated to examine the metal-dependence of BrxU (Figure 3C). φGeo gDNA was cleaved in the presence of Mg^2+^ and Mn^2+^ at ≥10^–4^ M, followed by both Fe^2+^ and Fe^3+^ allowing cleavage at ≥10^–3^ M, and Co^2+^ and Ni^2+^ at ≥10^–2^ M (Figure 3C). Ca^2+^, Cu^2+^ and Zn^2+^ did not support BrxU-dependent DNA cleavage (Figure 3C). As ATP was a required co-factor, a range of nucleotides were tested to examine co-factor promiscuity within BrxU. All the nucleotides (ATP, GTP, CTP, TTP and UTP) supported BrxU-dependent DNA cleavage of φGeo gDNA, with ATP as preferred nucleotide (Figure 3D). Non-hydrolysable analogues ATP-γ-S and GTP-γ-S prevented nucleolytic activity, and higher concentrations of GTP and CTP inhibited BrxU (Figure 3D). As per other GmrSD homologues (30), deoxynucleotides were also tested and all deoxynucleotides supported BrxU-mediated DNA cleavage (Figure 3E). Deoxynucleotide-dependent activity occurred in a concentration-dependent manner that did not show inhibition at higher concentrations, as seen for GTP and CTP (Figure 3D, E).

Next we aimed to determine whether the required nucleotides were indeed hydrolysed by BrxU and whether hydrolysis altered in the presence of substrate DNAs. Nucleotide hydrolysis was monitored by detection of released inorganic phosphate (P_i_), using BrxU-susceptible φBGP gDNA as substrate unless stated otherwise (Figure 3F). All nucleotides and deoxynucleotides were hydrolysed by BrxU, generating greater levels of P_i_ than the no nucleotide and non-hydrolysable nucleotide controls (Figure 3F). Generated P_i_ levels were greater for the nucleotide than corresponding deoxynucleotide co-factors, adenine and thymine were the preferred bases for both the nucleotide and deoxynucleotide sets, and ATP was the clear preferred substrate (Figure 3F). P_i_ production remained high in the absence of DNA and was not stimulated in the presence of unmodified (Lambda) or another modified (φGeo) gDNA; P_i_ production was also reduced in the absence of magnesium, or by the addition of EDTA (Figure 3F).

We then performed analytical size exclusion chromatography (SEC) to examine potential conformational changes within BrxU in the presence of nucleotides, with or without magnesium (Figure 3G). Without nucleotide or metal co-factors, BrxU eluted at a volume that indicated it is a dimer in solution (Figure 3G). When ATP was added, but not magnesium, BrxU eluted as a monomer, suggesting dissociation upon nucleotide binding (Figure 3G). Magnesium alone did not cause dissociation (Figure 3G). In contrast to ATP, which caused magnesium-independent dissociation of BrxU, all other nucleotides and deoxynucleotides only induced BrxU dissociation when co-incubated with magnesium, and the dissociation was often not complete (Figure 3G). The non-hydrolysable ATP and GTP analogues behaved in the same way as the hydrolysable counterparts, with ATP-γ-S inducing magnesium-independent dissociation, and GTP-γ-S requiring magnesium to do the same (Figure 3G). Finally, whilst AMP could not induce dissociation either with or without magnesium, ADP caused magnesium-dependent dissociation, suggesting the β-phosphate is needed for dissociation, as well as the specific base interactions (Figure 3G).

Here, we have shown that BrxU is a promiscuous enzyme that uses a range of nucleotide and metal co-factors to recognise and cleave DNA that contains any of at least three different modifications, and this activity is dependent on the initial nucleotide-driven dissociation of BrxU dimers, and subsequent nucleotide hydrolysis. The ability of BrxU to target modified phage DNA provides a neat complement to BREX-mediated phage resistance, which will target non-modified phage DNA (25). Together, BrxU and BREX comprise a phage defence island that delivers two independent layers of defence from phage infection.

### BrxU forms an interlocked dimer

**Figure 4.**
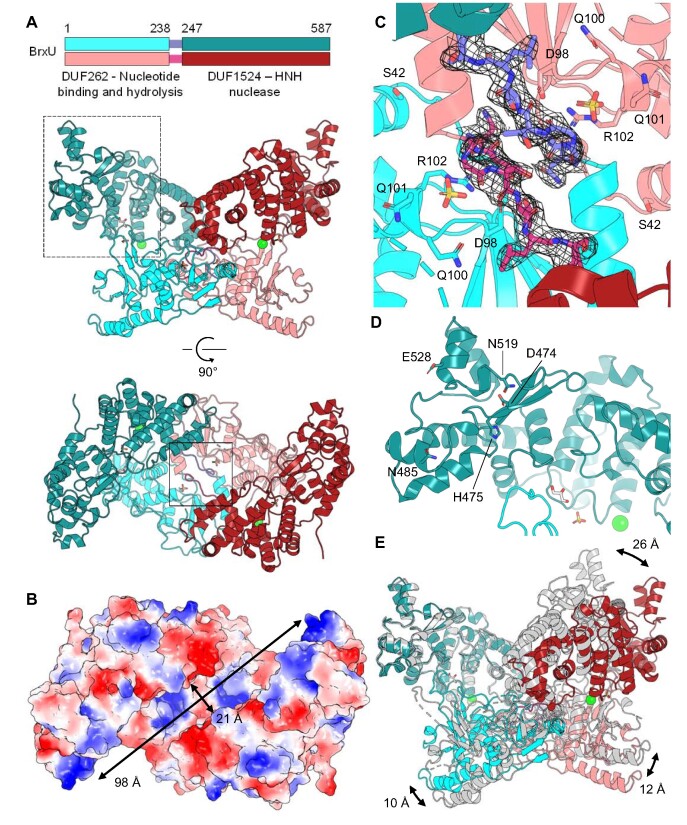
BrxU forms an intertwined dimer. (**A**) Cartoon overview of the BrxU dimer, shown as orthogonal views. Each protomer in the dimer is coloured either in shades of cyan or pink, with differing colours for domains and linkers as indicated. Numbers show amino acid positions. Chloride ions are represented as green spheres, sulphate ions are represented as sticks, and glycerol molecules are represented as sticks with white carbon backbones. (**B**) Electrostatic surface potential shows patches of blue electropositivity throughout the surface of a cleft running diagonally across the BrxU dimer. (**C**) Solid boxed region of (A), containing the proposed nucleotide binding site occupied by a sulphate ion and an α-helix and linker from the opposing protomer. The linker regions are shown with a 2*F*_o_ – *F*_c_ electron density map contoured to 1.0σ, which demonstrates the intertwined nature of dimerisation. Selected amino acids within the RLFDS and DGQQR motifs are shown as sticks. (**D**) Dashed boxed region of (A), close-up of the DHIYP motif region within the DUF1524 HNH nuclease C-terminal domain of the first protomer. An extended loop from the N-terminal domain of this protomer can be seen bridging to the C-terminal domain through a glycerol molecule. (**E**) Overlay of the BrxU dimer with a second BrxU dimer structure solved to 2.85 Å, coloured in light grey, shows inherent flexibility in domain position. The alignment was performed through the C-terminal domain of the first protomer (teal), to allow better visualisation of the relative movements of the other three domains in the dimer.

In order to better understand the biochemical data, the dimeric structure of BrxU was solved to 2.12 Å (Figure 4A and Supplementary Table S2). The N-terminal DUF262 domain of BrxU is connected to the C-terminal DUF1524 domain by a flexible linker. These linkers entwine in the observed dimer, and then a loop from each N-terminal domain connects with the corresponding protomeric C-terminal domain (bridged by a glycerol molecule in the structure), such that the dimer is, in effect, two interlocked circles. The relative rotation between N- and C-terminal domains is easily observed from a top-down view (Figure 4A), and generates a cleft that runs diagonally across the dimer. This cleft is ∼21 Å wide, and surface electrostatics show clear patches of electropositivity throughout, with the N-terminal domains as the floor and the C-terminal domains forming the walls (Figure 4B).

Bioinformatic analyses of GmrSD homologues identified RLFDS and DGQQR motifs related to nucleotide binding and hydrolysis, respectively, within the N-terminal DUF262 domains, and a DHIYP motif as part of a conserved HNH nuclease within the C-terminal DUF1524 domains (28). Previous structural modelling suggested that the γ-phosphate of a nucleotide substrate would be positioned by the equivalent of BrxU D98, Q100, Q101 and R102 (28). Within our structure, the position of the γ-phosphate has been taken by a sulphate ion that is bound by R102, and nucleotide binding has been prevented by proximity of an α-helix and linker from the opposing protomer that fill the corresponding space where a nucleotide might bind, also blocking S42 of the RLFDS motif (Figure 4C). These entwined linkers between the N- and C-terminal domains are shown with electron density from a 2*F*_o_ – *F*_c_ map contoured to 1.0σ (Figure 4C). Our new understanding of the BrxU dimers raises the possibility of a link between nucleotide binding and dimer dissociation at a structural level, wherein the nucleotide displaces the other protomer and promotes disentanglement of the linker regions, to generate monomers prior to nucleotide hydrolysis.

Within the C-terminal DUF1524 domain, D474 and H475 of the BrxU DHIYP nuclease motif are positioned on the surface of the DUF1524 domain to form part of the cleft wall within BrxU (Figure 4D). A glycerol molecule bridges the DUF1524 with a loop extending from the DUF262 domain, and an additional sulphate ion and chloride ion were observed bound by looped regions of DUF1524 (Figure 4D). Despite setting crystals in the presence of AMP-PnP, no nucleotide ligand was detected in the structure. The hypothesis had been that AMP-PnP would stabilise a shift from BrxU dimers to the monomeric form, to aid crystallisation. Upon subsequent analytical SEC analysis, however, we observed that whilst ATP and ATP-γ-S caused a concerted shift to monomers (Figure 3G), AMP-PnP unexpectedly did not (Supplementary Figure S4). It therefore makes sense that our crystals contain BrxU dimers, without bound nucleotide. Using ATP-γ-S in future studies may yield a nucleotide-bound monomeric structure.

Whilst using the first BrxU structure to analyse datasets from a range of BrxU crystals, a second dimeric structure with a differing conformation of BrxU was solved, this time to 2.85 Å (Figure 4E and Supplementary Table S2). Our models described above for DNA-binding and dimer dissociation rely upon a great deal of flexibility. By aligning the two solved structures, domain movements were visualised within the BrxU dimer (Figure 4E). The overall alignment provides an RMSD of 3.85 Å, indicating a poor average superposition (Figure 4E). By aligning the lower resolution structure through one C-terminal domain of the higher resolution protomer, (which as independent domains aligned well at 0.58 Å), distinct relative movements of each of the other domains of the lower resolution structure can be observed (Figure 4E). In this manner, we can see a 10 Å movement of the protomeric N-terminal domain, and corresponding shifts of 12 and 26 Å for the N-terminal and C-terminal domains of the second protomer, respectively (Figure 4E). This level of movement confirms the flexibility of the BrxU dimer, and gives rise to an hypothesis that the 21 Å cleft might widen to accommodate a substrate DNA, with backbone phosphates supported by loops of the DUF1524 domain, as was seen for the observed sulphate and chloride ions (Figure 4D).

### BrxU-mediated phage resistance requires a multi-step process

To generate structure-function information, we mutated key residues in BrxU. Based on the alignment with Eco94GmrSD (Supplementary Figure S1), our structural information (Figure 4) and past bioinformatic studies (28), we generated 11 single point mutants and one double mutant of BrxU. To optimise over-expression and purification, mutants were made in pBAD30-*his_6_-brxU*^+^.

**Figure 5.**
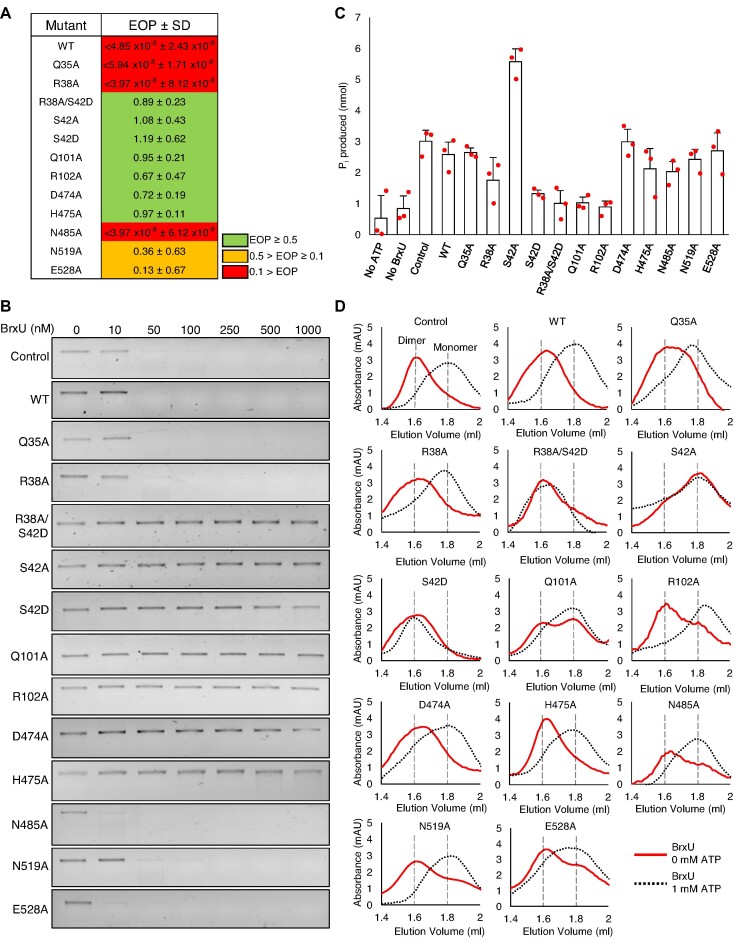
Analysis of BrxU mutants shows a complex reaction cycle. (**A**) EOP values for His_6_-BrxU and mutants against phage φGeo. Values are mean EOPs from triplicate data, shown with standard deviation. Values presented with < extended below the range of this assay and no plaques were observed. (**B**) DNA cleavage assays with His_6_-BrxU WT and mutants. φGeo gDNA was incubated with a gradient of BrxU concentrations at 37°C for 60 min, in the presence of 10 mM MgSO_4_ and 1 mM ATP, and resolved via agarose gel electrophoresis at 120 V for 45 min. Control sample is untagged BrxU expressed from pSAT1-LIC-*brxU*^+^. WT and mutants are expressed from pBAD30-*his_6_*-*brxU*^+^ and its derivatives. Data shown are representative of triplicate experiments. (**C**) Hydrolysis of ATP by His_6_-BrxU WT and mutants. Control sample is untagged BrxU expressed from pSAT1-LIC-*brxU*^+^. WT and mutants are expressed from pBAD30-*his_6_*-*brxU*^+^ and its derivatives. Presented data are the mean and standard deviations from triplicate experiments, with data points overlaid. (**D**) Analytical size exclusion analysis of His_6_-BrxU WT and mutants. 10 μl samples of 500 nM BrxU and 10 mM MgSO_4_, with and without 1 mM ATP, were resolved at 0.175 ml/min. Control sample is untagged BrxU expressed from pSAT1-LIC-*brxU*^+^. WT and mutants are expressed from pBAD30-*his_6_*-*brxU*^+^ and its derivatives. Traces in red represent samples that contained 0 mM ATP. Dashed traces in black represent samples that were pre-incubated at 37°C with 1 mM ATP prior to loading. Traces are representative of triplicate data, and relative elution volumes for the dimeric and monomeric forms of BrxU are indicated by dashed grey lines.

Following the generation of the BrxU mutant constructs, they were first tested for functionality in EOP assays (Figure 5A). The double mutant R38A/S42D and the single mutants S42A, S42D, Q101A, R102A, D474A and H475A were no longer phage-resistant, as the BrxU-dependent impact on phage EOPs was ablated (Figure 5A). In contrast, mutants N519A and E528A remained phage-resistant, although the impact on EOP had been reduced to less than a 10-fold change (Figure 5A). Mutants Q35A, R38A and N485A were as functionally phage-resistant as WT BrxU (Figure 5A).

The WT and mutant BrxU constructs where then used to over-express and purify pure proteins (Supplementary Figure S5A), which were tested for the ability to degrade φGeo gDNA (Figure 5B). The His_6_-BrxU WT protein degraded DNA as well as the untagged BrxU control (Figure 5B). Consistent with the EOP phenotypes, the double mutant R38A/S42D and single mutants S42A, S42D, Q101A, R102A, D474A and H475A lacked enzymatic activity and did not digest the DNA (Figure 5B). Also consistently, mutants Q35A, R38A and N485A digested the DNA as per WT (Figure 5B). Surprisingly, whilst mutants N519A and E528A had a minimal ability to restrict *in vivo* as shown by EOP (Figure 5A), both mutant proteins readily degraded the DNA substrate *in vitro* (Figure 5B).

The ability of the BrxU mutants to mediate nucleotide hydrolysis was then determined in the presence of ATP and magnesium (Figure 5C). Again, His_6_-BrxU WT behaved similarly to the untagged BrxU control (Figure 5C). Non-phage-resistant mutants S42D, R38A/S42D, Q101A and R102A all showed reduced production of P_i_. Unexpectedly, whilst mutant S42A was non-phage-resistant by EOP (Figure 5A), and no longer digested DNA *in vitro* (Figure 5B), the level of P_i_ production was increased nearly 2-fold over WT (Figure 5C). Phage-resistant mutants Q35A, R38A, and N485A had approximately the same P_i_ production levels as WT (Figure 5C). Weakly-phage-resistant mutants N519A and E528A also showed similar P_i_ production levels as WT (Figure 5C). Finally, mutants D474A and H475A, part of the DHIYP motif in the C-terminal domain (Figure 4D), were both non-phage-resistant by EOP (Figure 5A) and unable to digest DNA (Figure 5B), but showed WT levels of P_i_ production (Figure 5C). This demonstrates that nucleotide hydrolysis can occur independently within the N-terminal domain, whilst DNA cleavage activity is associated with the C-terminal domain.

Our findings reveal that nucleotide binding and hydrolysis must occur before BrxU-mediated DNA cleavage, as P_i_ production by the N-terminal nucleotide hydrolysis domain was seen in the absence of a functional C-terminal nuclease domain, but no DNA cleavage occurred without functional nucleotide binding and hydrolysis. We speculate that nucleotide binding leads to BrxU dissociation, and that subsequent hydrolysis allows re-association of BrxU monomers, potentially concomitantly with binding to substrate DNA, followed by cleavage. If this were the case, rounds of dissociation and re-association would allow BrxU to rapidly hop on and off the DNA substrate.

As BrxU dissociation is likely required for phage resistance, we wanted to understand how the mutations impacted the ability of BrxU to shift from dimer to monomer. The mutant BrxU proteins were therefore examined by analytical SEC in the presence of magnesium, with or without ATP (Figure 5D). The His_6_-BrxU WT protein shifted from dimer to monomer form in the presence of ATP, as observed for the untagged BrxU control (Figure 5D). All mutations in the C-terminal domain (D474A, H475A, N485A, N519A and E528A) behaved similarly to WT (Figure 5D). Phage-resistant mutants Q35A and R38A also behaved as WT (Figure 5D).

Non-phage-resistant mutant Q101A was equally distributed between dimeric and monomeric forms prior to addition of ATP, and both Q101A and R102A dissociated in the presence of ATP, indicating nucleotide binding (Figure 5D). As these mutants could clearly bind but not then hydrolyse the nucleotide (Figure 5C), this both fits their suggested role in positioning the γ-phosphate (Figure 4C), and indicates that nucleotide binding is sufficient for dissociation. Double mutant R38A/S42D and single mutant S42D did not dissociate in the presence of ATP, which explains their lack of phage resistance (Figure 5A/D). Finally, non-phage-resistant mutant S42A formed monomers even in the absence of ATP (Figure 5D). The varied phenotypes of each mutant were summarised to provide an overview (Supplementary Figure S5B).

S42 lies within the dimerisation interface (Figure 4C) and is clearly vital for mediating the switch from dimer to monomer. S42A being permanently locked as a monomer would explain the increased rate of nucleotide hydrolysis leading to higher P_i_ production (Figure 5C). The fact that the S42A monomeric mutant is non-phage-resistant further demonstrates that nucleotide binding and hydrolysis is not sufficient for DNA cleavage, but that there must be rounds of both dissociation and re-association for BrxU-mediated DNA cleavage to occur. These data indicate that a complex cycle of events occurs to control the phage-resistance activity of BrxU (Supplementary Figure S6), as part of the wider phage defence provided by pEFER.

## DISCUSSION

**Figure 6.**
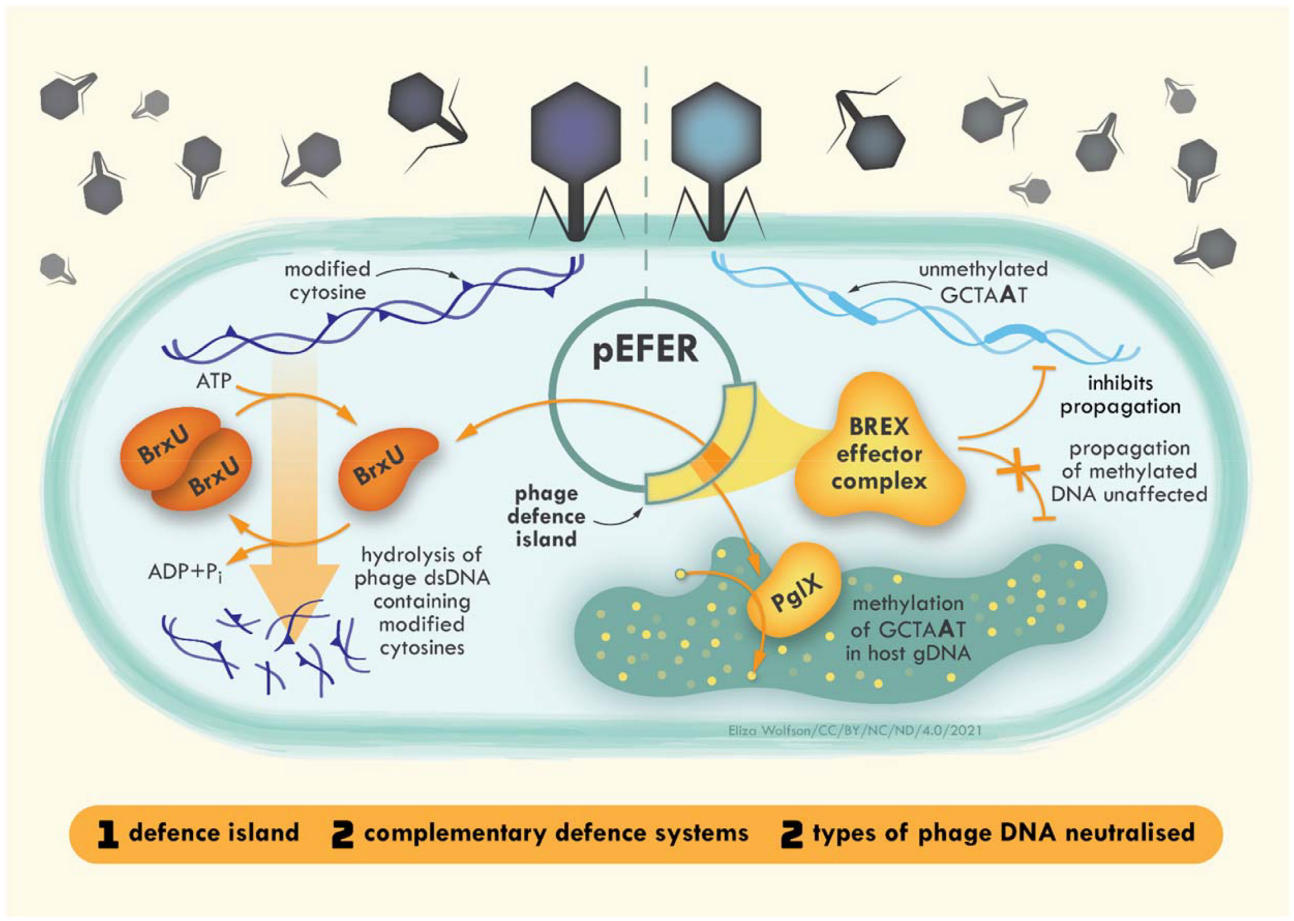
Encoding multiple phage defence systems as a defence island manifests a complementary bacterial immune system capable of suppressing multiple phage types. Phage genomes that contain modified cytosines are hydrolysed by BrxU. BREX effector proteins recognise unmethylated non-palindromic BREX motifs in phage gDNA and prevent phage propagation. Methylation of this motif on host DNA by PglX protects the host from BREX effectors.

Plasmid pEFER encodes a defence island that uses at least two complementary systems to protect bacteria from phage attack, one recognising specific non-modified sequence motifs within injected phage DNA (BREX), and a second recognising DNA with specific modifications (BrxU) (Figure 6). As DNA modifications prevent BREX activity (25), phages that have evolved to have modifications avoid targeting by BREX, but then can become susceptible to BrxU (Figure 6). This ‘belt and braces’ approach ensures better protection for the bacterial host.

Whilst pEFER carries a type I BREX system (17), the core genes are preceded by non-canonical upstream genes *brxS*, *brxT* and *brxR* (Figure 1A). Of particular interest, BrxR contains a WYL domain, which has been associated with a wide range of phage-resistance mechanisms as a likely ligand-dependent transcriptional regulator (57). The function of these additional genes is currently unclear and remains to be determined through systematic deletion, tests against our suite of phages and examination of DNA modification through PacBio sequencing. This will help to identify the minimal requirement for phage defence. The pEFER BREX provided protection against five of the tested phages (Figure 2B). Of course, the BrxU-sensitive phages might have the pre-requisite GCTAAT sequences in order to be targeted by BREX, but the DNA modifications making them BrxU-sensitive would prevent BREX activity (25). It will be interesting to sequence the genomes of all our phages in due course, to identify GCTAAT motifs. Any phage that has GCTAAT motifs, but is both BREX-resistant by EOP assay, and is resistant to *in vitro* digestion by BrxU, would by definition encode a BREX inhibitor, as recently demonstrated for Ocr from phage T7 (58). Thirteen phages were BrxU-sensitive. Inhibition of the BrxU homologue GmrSD was observed with IPI*, a protein co-injected with T4 DNA (29,30,59). If a similar inhibitor was used by any of our phages, this would have been identified by the phage having a high EOP value (Figure 2D) but then having gDNA digested during *in vitro* BrxU assays (Figure 3A and Supplementary Figure S2). Such a comparison did not identify any likely candidates with a BrxU inhibitor within our phage suite.

BrxU is a fused form of the bipartite GmrS/GmrD type IV restriction system (24,29). Previous characterisation of the fused homologue Eco94GmrSD showed an ability to cleave glc-5hmC and 5hmC modified DNAs, using Mg^2+^ or Mn^2+^, and a limited set of nucleotides (30). In contrast, BrxU can utilise a much wider wide range of metal and nucleotide co-factors, and cleaves DNA containing one of at least three DNA modifications, 5mC, 5hmC or glc-5hmC (Figure 3). As BrxU has been shown to hydrolyse a wide range of nucleotides *in vitro* (Figure 3F) it might be expected to be toxic due to depletion of the cellular nucleotide pool. This has previously been considered for type I R-M systems, and comparing the overall cellular ATP turnover rates to the effect of these enzymes operating at maximum indicated they would use up only 0.2% of the available ATP (60). Accordingly, we have not observed toxicity from BrxU even when over-expressed. Nevertheless it would still be of interest to examine the kinetics of BrxU-dependent nucleotide turnover and whether it is coupled to DNA binding and cleavage. The full range of DNA modifications recognised by BrxU also remains to be investigated, and it is worth noting that strains containing the pEFER defence island have the N6mA BREX modifications without impact from BrxU, suggesting BrxU does not recognise N6mA. As per other type IV restriction enzymes (61), it is also likely that there will be a specific sequence motif required for BrxU cleavage as a result of recognising modifications. Further functional and structural information will be needed to understand the basis of BrxU modification recognition and sequence preference. The unrelated type IV enzyme AbaSI uses an SRA-domain to recognise modified DNA substrates in a conserved pocket (61), but no such obvious pocket was observed for BrxU (Figure 4).

Strikingly, we have shown that nucleotide binding unexpectedly shifts BrxU dimers towards a monomeric state (Figure 3). Our BrxU structures are the first for the GmrSD family, providing the highest resolution detail for the widespread DUF262 and DUF1524 domains (28) (Figure 4). The structures reveal how nucleotide binding is blocked in the dimer form (Figure 4C) and reveals inherent flexibility that would allow for cycles of dimer separation (Figure 4E). Further functional and structural characterisation of BrxU monomers and dimers binding to co-factors and DNA substrates will be needed to illuminate multiple aspects of BrxU biochemistry. These will build on structural and mutagenesis studies that have identified specific steps of a complex reaction cycle that so far includes nucleotide binding, monomerisation, nucleotide hydrolysis, dimerisation (perhaps in concert with modified DNA recognition), and DNA cleavage (Supplementary Figure S6). Further study will show how BrxU might transfer between cleavage sites, and how it is possible for BrxU to be so promiscuous. These types of developments will lead to a better theoretical appreciation of how biological systems are able to utilise a range of nucleotides, and to the design of selective reagents to simplify the mapping of epigenetic DNA modifications.

Plasmid pEFER provides the host with antibiotic-resistance and phage-resistance, both of which will be advantageous in a dynamic environment. Our investigation of how this multidrug-resistant plasmid protects its bacterial host from phage infection has highlighted the interplay of multiple complementary phage-resistance systems encoded by a defence island on the plasmid. The recently discovered prevalence of defence islands (14,15) may well lead to the discovery of even more diverse and complex interplays of defensive systems. It is likely that these will be thwarted by counter-defence islands, as seen for anti-CRISPRs (62,63). The biochemical characterisation of reciprocal islands could expand our phage-derived arsenal of biotechnological tools even further.

## DATA AVAILABILITY

The crystal structures of BrxU have been deposited in the Protein Data Bank under accession numbers 7P9K (native 2.12 Å structure) and 7P9M (native 2.85 Å structure). All other data needed to evaluate the conclusions in the paper are present in the paper and/or Supplementary Data.

## Supplementary Material

gkab906_Supplemental_File
