# Supplementary material for: The phage defence island of a multidrug resistant plasmid uses both BREX and type IV restriction for complementary protection from viruses
Source: Nucleic Acids Res. 2021 Oct 18;49(19):11257–73. doi: 10.1093/nar/gkab906 (PMC8565348; doi:10.1093/nar/gkab906)
Supplement: gkab906_Supplemental_File [file gkab906_supplemental_file.pdf]

# Supplementary Data

**The phage defence island of a multidrug resistant plasmid uses both BREX and type IV restriction for complementary protection from viruses**

David M. Picton[a], Yvette A. Luyten[b], Richard D. Morgan[b], Andrew Nelson[c], Darren L. Smith[c], David T.F. Dryden[a], Jay C. D. Hinton[d], Tim R. Blower[a,*]

[a]Department of Biosciences, Durham University, Stockton Road, Durham, DH1 3LE, UK.

[b]New England Biolabs, 240 County Road, Ipswich, MA 01938, USA.

[c]Department of Applied Sciences, University of Northumbria, Newcastle Upon Tyne, NE1 8ST, UK.

[d]Institute of Infection, Veterinary and Ecological Sciences, University of Liverpool, Liverpool, L69 7ZB, UK.

[*]To whom correspondence may be addressed. Email: timothy.blower@durham.ac.uk, tel: +44(0)1913343923.

Keywords: BREX, Type IV Restriction Enzyme, Bacteriophage Defence, GmrSD, Defence Island

# Supplementary Figure S1

```
                          N-terminal                         C-terminal
                        DUF262 Domain      Linker Region   DUF1524 Domain

BrxU                1  MYQAGGTIRSLLDKVAEQEYLLPAIQREFVWR-PEQICRLFDSLLQG---   46
Eco94GmrSD          1  MKSETLTIQQIFQN--QRQYRVPFYQRAYVWTQRNQWSALLEDIFEKAQS   48
Conservation:          Mbp.s.TIpplhpp..pppY.lPhhQR.@VWp..pQht.Lh-slhp....
Predicted 2°:                 hhhhhhhhh ssss            hhhhhhhhhhhhhhhhhh

BrxU               47  ---------YPFGTFLFWKIKPENRDSYQFYQFMQHYHERDNYHCENVTQ   87
Eco94GmrSD         49  RLSGTKPTPHFLGAVVLEPQLK--------------------------NS   72
Conservation:          .........@.hGhhlhb.bb..........................sp
Predicted 2°:          hh            ssss              sss

BrxU               88  LPEREFIAVLDGQQRITALNIGLRGSFAWKLTGKWWSNDDAFPVRRLHLN  137
Eco94GmrSD         73  LLGVDTIHIIDGQQRLTTLQYILASIRLSLRATGLSELEGLVLTCLKNTN  122
Conservation:          L...-hIhllDGQQRlThLph.L.t..h.b.hs.h.p.-shh.h..bphN
Predicted 2°:               sssss  hhhhhhhhhhhhhhhhhhhhh hhhhhhhhhhhhhhhh

BrxU              138  LLSKPDLET-----------GSMYDFEFLTDDKASLDAS-----------  165
Eco94GmrSD        123  EATMRNKKVECFKLWPTFRDQTHFIQSLNVDNIDDLRNVFSDSFTQHGTL  172
Conservation:          bhob.sbch...........oh@.bph.hDsbssLcss...........
Predicted 2°:           h                   hhhhhhhh         hh

BrxU              166  ----------EQYWFRVGRIMEEEEDALIDEVADDARLSSEQRKEARSTL  205
Eco94GmrSD        173  RKHFNHPPSLEALWFFTEA---------------FIKWIKIENHSPQENA  207
Conservation:          ..........E.hWF.h.................h+h.pbpp+psppsh
Predicted 2°:          hh    hhhhhhhhhhhhhhh    hhhhhhhhhhhhhhhhhhhhhhhhh

BrxU              206  RHLYRTIHDKDKISFYEESDQSLERVLNIFIRMNSGGTTLSYSDLLLSIA  255
Eco94GmrSD        208  VALIEAVLTDLKLVSIFLEAE--DDAQIIFETLNGRGAELHATDLIRNYI  255
Conservation:          .hLhchlhsc.Kls.hbbpsp..-chb.IFbphNt.GhpLphoDLl.shh
Predicted 2°:          hhhhhhhhh   sssssss    hhhhhhhhhhh        hhhhhhhh

BrxU              256  ---------------VAQWSSLD-------------AREEIHALVDEMNR  277
Eco94GmrSD        256  FMCAEHENINAIELYENEWKIFEDKYWSEKQRRGRINKPRMEWLVHATLQ  305
Conservation:          ...............spWp.h-.............s+.chchLVc.h.p
Predicted 2°:          hhhh    hhhhhhhhhhhhhhhhhhhhhhhhhhhhhhhhhhhhhhhhhh

BrxU              278  VGDGFNVSKDLVLKAGLMLSDIGSVGFKVENFNKENMAILEKNWTPIRD-  326
Eco94GmrSD        306  SERQREIDLSHLYNEYRDYVSKDLPSQRADLQ----VKRLKQYASQYKEL  351
Conservation:          s.c..plsbshlhp....hssbs.stb+h-.b....h..Lcp.ho.h+-.
Predicted 2°:          hhh     hhhhhhhhhhhhh    hhhhhhhhhhhhhhhhhhhhhhhhh

BrxU              327  -------ALLLSMQLLASFGFNAQNLRATSAILPLAYYLHHRKLTASYLS  369
Eco94GmrSD        352  VGGFGTTPISHFGHRIAAYDVT--------TLYPLALFISIANIADDEK-  392
Conservation:          .......sl.h..p.lAt@shs........hlhPLAh@lph.plhssbb.
Predicted 2°:          h      hhhhhhhhhhh    hhh  hhhhhhhhhhhhhh hhhhhhh

BrxU              370  RVEYAVDRECIRNWLIRSLLKASGIWGSGLDTLLTMLRSDIKQSGDTGFP  419
Eco94GmrSD        393  ----AAMYNDLVSYVVRRSVC--GLTPKNYNNVFMNVLRHLSKTEISSVE  436
Conservation:          ....Ah..psl.s@llRp.l...Glhspshsslhh.l.pclppo..oth.
Predicted 2°:              hhhhhhhhhhhhhhhhhhhhh  hhhhhhhhhhhhhhhhhhh hhh

BrxU              420  LAKIEATMQQRGKSLRFDPEEISELAQL------DYGNPRTFALLTLLF-  462
Eco94GmrSD        437  LRNILNSLNGEASRWP-GDSEFLNACINAPLYPGRLDAPKMRSMLTELER  485
Conservation:          L.pIbsohp.ctpph..sspEh.phtb......chssP+h.thLTbLb.
Predicted 2°:          hhhhhhhhhhhhh     hhhhhhhhhh          hhhhhhhhhhhh

BrxU              463  ----------PGFDFSRHFHVDHIYPKGLFTRNKLA--------------  488
Eco94GmrSD        486  ELCRQVKTEKPDVPNLSNLDIDHLMPQSWYSCWPLENGHMVTNSDATVMN  535
Conservation:          ..........Pshs..pphclDHlhPpth@o...L...............
Predicted 2°:          hhhhh              sssssss                    hhhhhh

BrxU              489  -------KVGVPAEQLDELIEASNKLPNLQLLEGTINNQKRQKMPHEWYA  531
Eco94GmrSD        536  QIVLSGTDLTPEQLLVRKRQQAIATLGNLTLLNLSVNRSVQNAVFLKKRD  585
Conservation:          .......clss..bblcc.bpA.spLsNLpLLp.olNpp.pp.h.hcb.s
Predicted 2°:          hhh         hhhhhhhhhhhhhh        hhhhhh    hhhhhh

BrxU              532  QQWPDVNARQAHLQSQAITSLPEQLNQFMDFYRERQETLLARIRTALQPA  581
Eco94GmrSD        586  ALIVHTN---LRLNIPLILKD--KWD---------ESEILERGKKLGEIA  621
Conservation:          .bhschN...h+Lp..hIhp...phs.........ppplL.R.+ph.p.A
Predicted 2°:          hhhh    hhhhhhhhh                  hhhhhhhhhhhhhhhhhhhhhhh

BrxU              582  SSVETE--  587
Eco94GmrSD        622  LKVWPKYD  629
Conservation:          .pVbsc..
Predicted 2°:          hhh
```

**Supplementary Figure S1.** Amino acid sequence alignment of BrxU and Eco94GmrSD. BrxU and Eco94GmrSD were aligned using PROMALS3D. Predicted domain architectures are indicated. RLFDS, DGQQR and DHIYP motifs are highlighted in yellow. Residues selected for mutation (Figure 5) are shown in red and underlined. Conservation of residues is denoted using the following key: conserved amino acid residues are shown in bold and uppercase letters; conserved aliphatic residues (I, V, L), shown as *l*; conserved aromatic residues (Y, H, W, F), shown as @; conserved hydrophobic residues (W, F, Y, M, L, I, V, A, C, T, H), shown as *h*; conserved alcohol residues (S, T), shown as o; conserved polar residues (D, E, H, K, N, Q, R, S, T), shown as p; conserved "tiny" residues (A, G, C, S), shown as t; conserved small residues (A, G, C, S, V, N, D, T, P), shown as s; conserved bulky residues (E, F, I, K, L, M, Q, R, W, Y), shown as b; conserved positively charged residues (K, R, H), shown as +; conserved negatively charged residues (D, E), shown as -; conserved charged residues (D, E, K, R, H), shown as c. Secondary structure (2°) prediction denoted using the following key; α-helices, h, β-strands, s.

# Supplementary Figure S2

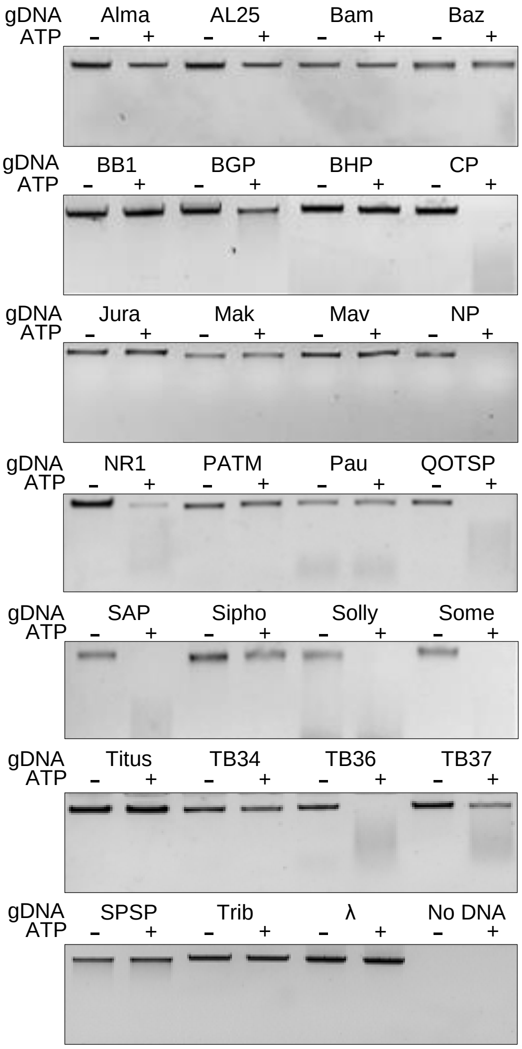


**Supplementary Figure S2.** Phage gDNA cleavage by BrxU. Phages with reduced EOP values in the presence of pBAD30-*his*$_6$-*brxU*$^+$ (**Figure 2D**) have their gDNA cleaved by BrxU *in vitro*. All lanes contain 500 nM BrxU and 10 mM $MgSO_4$, with and without 1 mM ATP. All phage genomes were tested in this manner. All samples were resolved in 1% agarose TAE at 120 V for 45 min. Data are representative of triplicate experiments.

# Supplementary Figure S3

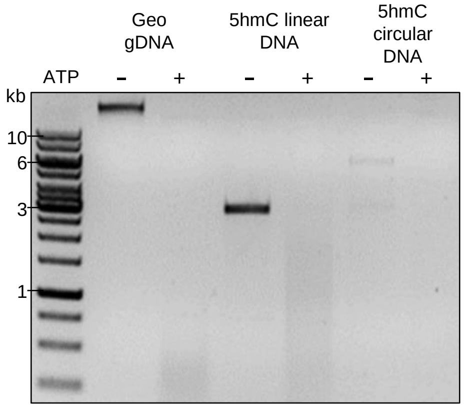


**Supplementary Figure S3.** BrxU cleaves both linear and circular modified DNA substrates. The 5hmC-containing linear DNA product (**Figure 3B**) was phosphorylated and ligated to produce a circular product. A faint band representing circular DNA can be seen at ~5 kb. Both linear and circular forms were degraded by BrxU. ϕGeo gDNA is shown as a positive control. All lanes contain 500 nM BrxU and 10 mM $MgSO_4$, with and without 1 mM ATP. All samples were resolved in 1% agarose TAE at 120 V for 45 min. Data are representative of triplicate experiments.

# Supplementary Figure S4

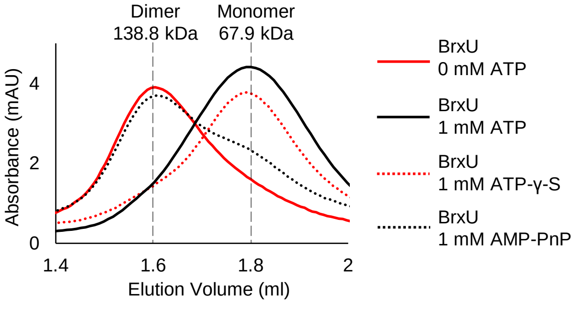


**Supplementary Figure S4.** BrxU does not fully dissociate when incubated with AMP-PnP. Analytical size exclusion analysis of untagged BrxU WT with a range of ATP analogues. 10 µl samples of 500 nM BrxU and 10 mM $MgSO_4$, with 1 mM NTP (or no NTP control), were resolved at 0.175 ml/min. Untagged BrxU was expressed from pSAT1-LIC-*brxU*$^+$. Traces are representative of triplicate data, and relative elution volumes for the dimeric and monomeric forms of BrxU are indicated by dashed grey lines.

# Supplementary Figure S5

**A**

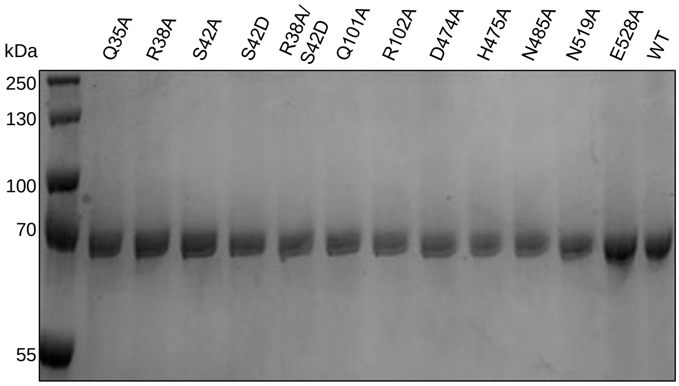


**B**

| Mutant | Resistant against phage ϕGeo | Digests ϕGeo gDNA | Hydrolyses ATP | Dimer in absence of ATP | Switches to Monomer in 1 mM ATP |
|---|---|---|---|---|---|
| WT | Y | Y | Y | Y | Y |
| Q35A | Y | Y | Y | Y | Y |
| R38A | Y | Y | Y | Y | Y |
| R38A/S42D | N | N | N | Y | N |
| S42A | N | N | Y – Increased hydrolysis | N - Monomer | Obligate monomer |
| S42D | N | N | N | Y | N |
| Q101A | N | N | N | Intermediate | Y |
| R102A | N | N | N | Y | Y |
| D474A | N | N | Y | Y | Y |
| H475A | N | N | Y | Y | Y |
| N485A | Y | Y | Y | Y | Y |
| N519A | Intermediate | Y | Y | Y | Y |
| E528A | Intermediate | Y | Y | Y | Y |

**Supplementary Figure S5.** Purified BrxU mutants expressed from pBAD30-*his*$_6$-*brxU*$^+$. (A) Samples resolved in 10% acrylamide at 180 V for 100 min. Each lane contains 100 µg of protein. (B) Summary of mutant phenotypes (**Figure 5**). Y=Yes, N=No.

# Supplementary Figure S6

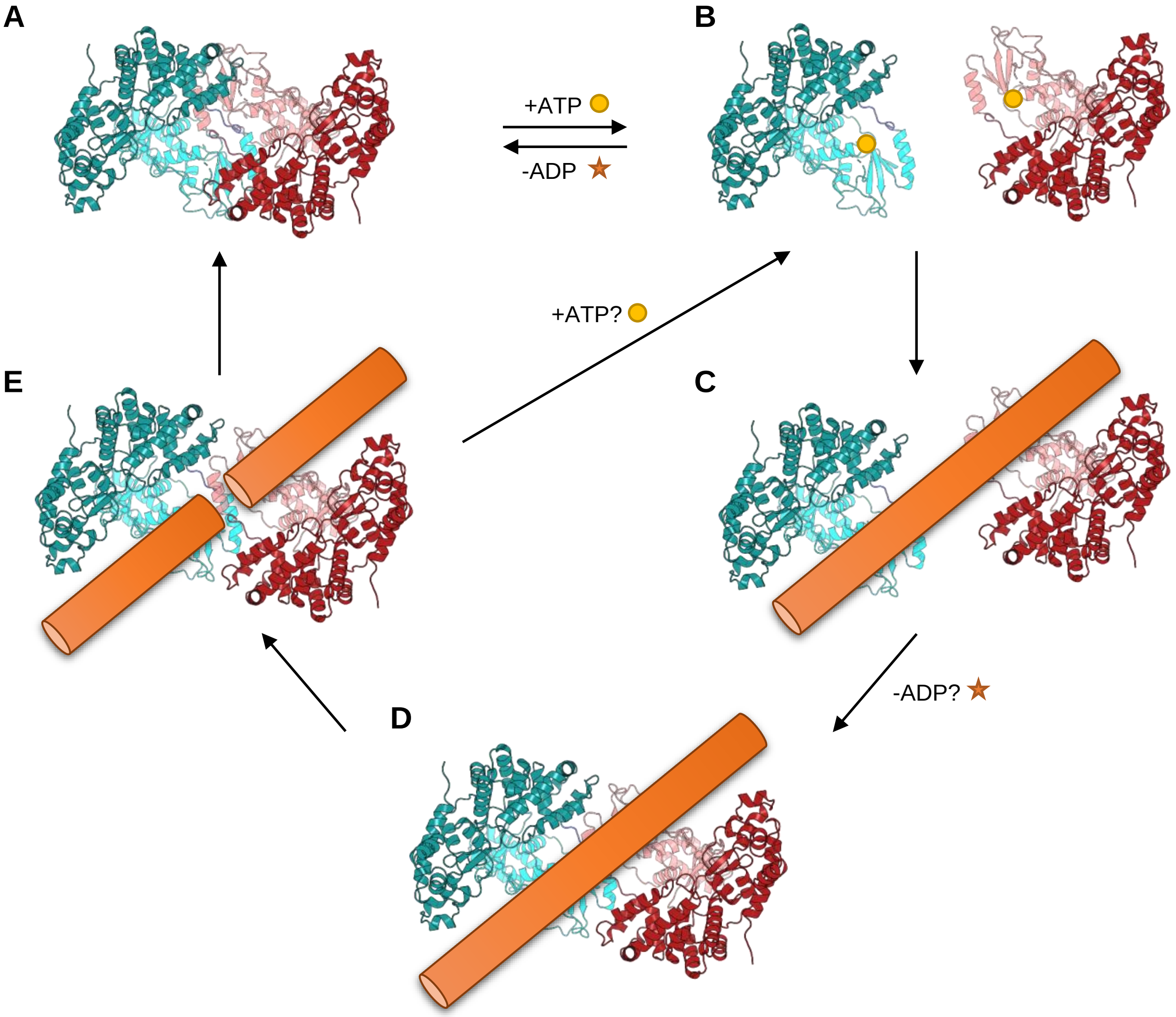


**Supplementary Figure S6.** BrxU undergoes a proposed multi-step reaction cycle. (A) In the absence of nucleotide, BrxU forms dimers. (B) Upon nucleotide binding, BrxU dissociates into monomers, and can re-associate with the release of ADP. (C) BrxU monomers bind double-stranded DNA substrates (orange cylinder). (D) Once a DNA modification is recognised, the BrxU monomers re-associate, with concomitant loss of ADP. It is currently unclear whether substrate DNA is bound by BrxU monomers or dimers, and when ADP is released. (E) The modified DNA is then cleaved by BrxU. The cleavage sequence remains to be uncovered, and whether BrxU cuts once or twice per dimer. Following DNA cleavage, BrxU dimers might either dissociate from the DNA (A) or bind ATP to cause complex dissociation, releasing the cleaved DNA and returning BrxU to monomeric form (B).

# SUPPLEMENTARY TABLES

**Supplementary Table S1. Annotation of pEFER (Genbank CU928144) ORFs with predicted function.**

| Gene | Size (bp) | Orientation | Start | End | BLASTp output and predicted function |
|---|---|---|---|---|---|
| EFER_p01 | 866 | Fw | 450 | 1316 | Replication initiation protein |
| EFER_p02 | 1218 | Fw | 1637 | 2855 | Hypothetical protein |
| EFER_p03 | 167 | Fw | 1840 | 2007 | AAA ATPase |
| EFER_p04 | 1205 | Fw | 2249 | 3454 | Plasmid partition protein ParA |
| EFER_p05 | 977 | Fw | 3451 | 4428 | Plasmid partition protein ParB |
| EFER_p06 | 1271 | Fw | 4510 | 5781 | Y family DNA polymerase SamB |
| EFER_p07 | 707 | Fw | 5781 | 6488 | lmpA |
| EFER_p08 | 167 | Fw | 6370 | 6537 | Hypothetical protein |
| EFER_p09 | 752 | Fw | 6557 | 7309 | RetA, putative reverse transcriptase |
| EFER_p10 | 416 | Rv | 6614 | 7030 | Hypothetical protein |
| EFER_p11 | 134 | Fw | 7054 | 7188 | Putative reverse transcriptase |
| EFER_p12 | 275 | Fw | 7461 | 7736 | IS1 family transposase |
| EFER_p13 | 503 | Fw | 7655 | 8158 | IS1 family transposon |
| EFER_p14 | 299 | Fw | 9008 | 9307 | Hypothetical protein |
| EFER_p15 | 968 | Fw | 9620 | 10588 | IS5 family transposase |
| EFER_p16 | 218 | Fw | 10781 | 10999 | Hypothetical protein |
| **EFER_p17** | **671** | **Fw** | **11390** | **12061** | **1S3 family transposase BrxS** |
| EFER_p18 | 179 | Rv | 12093 | 12272 | Hypothetical protein |
| **EFER_p19** | **704** | **Fw** | **12109** | **12813** | **Hypothetical protein BrxT** |
| **EFER_p20** | **926** | **Fw** | **12882** | **13808** | **WYL-domain transcriptional regulator BrxR** |
| **EFER_p21** | **599** | **Fw** | **13818** | **14417** | **Putative DUF1819 inner membrane protein BrxA** |
| **EFER_p22** | **593** | **Fw** | **14405** | **14998** | **Hypothetical protein BrxB** |
| **EFER_p23** | **3677** | **Fw** | **15017** | **18694** | **AAA ATPase BrxC** |
| **EFER_p24** | **1763** | **Fw** | **18711** | **20474** | **DUF262 DUF1524 restriction endonuclease BrxU** |
| **EFER_p25** | **3653** | **Fw** | **20490** | **24143** | **Methyltransferase PglX** |
| **EFER_p26** | **2657** | **Fw** | **24143** | **26800** | **Alkaline phosphatase PglZ** |
| **EFER_p27** | **2075** | **Fw** | **26817** | **28892** | **ATP dependent Lon-like protease BrxL** |
| EFER_p28 | 746 | Rv | 28885 | 29631 | IS110 family transposase |
| EFER_p29 | 179 | Rv | 29000 | 29179 | IS3 transposase |
| EFER_p30 | 2885 | Rv | 29749 | 32634 | Tn3 family transposase TnpA |
| EFER_p31 | 623 | Fw | 32760 | 33383 | Recombinase |
| EFER_p32 | 296 | Fw | 33393 | 33689 | Transposase element YahA |
| EFER_p33 | 665 | Fw | 33686 | 34351 | IS3/ IS1133 family transposase |
| EFER_p34 | 3164 | Rv | 34376 | 37540 | Tn3 family transposase TnpA |
| EFER_p35 | 593 | Fw | 37509 | 38102 | Tn3 resolvase TnpR |

| | | | | | |
|---|---|---|---|---|---|
| EFER_p36 | 860 | Fw | 38285 | 39145 | Broad spectrum beta-lactamase |
| EFER_p37 | 593 | Fw | 39261 | 39854 | IS4 family transposase |
| EFER_p38 | 416 | Rv | 39864 | 40280 | Tetracycline efflux protein TetD |
| EFER_p39 | 668 | Fw | 40293 | 40961 | Tetracycline efflux protein TetE |
| EFER_p40 | 1253 | Rv | 41074 | 42327 | Tetracycline efflux protein TetA |
| EFER_p41 | 626 | Fw | 42358 | 42984 | Tetracycline repressor protein TetR |
| EFER_p42 | 686 | Rv | 42962 | 43648 | Transcriptional regulator ArsR |
| EFER_p43 | 497 | Rv | 43656 | 44153 | Hypothetical protein |
| EFER_p44 | 50 | Rv | 44305 | 44355 | Antibiotic monoxygenase |
| EFER_p45 | 1205 | Fw | 44799 | 46004 | Glutamate permease JemA |
| EFER_p46 | 1208 | Rv | 46370 | 47578 | IS4 family transposase |
| EFER_p47 | 182 | Fw | 47886 | 48068 | Streptomycin kinase StrA |
| EFER_p48 | 803 | Fw | 47864 | 48667 | Streptomycin kinase StrB |
| EFER_p49 | 182 | Fw | 47886 | 48068 | Streptomycin kinase StrB |
| EFER_p50 | 566 | Fw | 49475 | 50041 | IS110 family transposase |
| EFER_p51 | 242 | Rv | 49963 | 50205 | AAA ATP-binding transposase |
| EFER_p52 | 587 | Rv | 50260 | 50847 | IS3 family transposase |
| EFER_p53 | 416 | Rv | 51383 | 51799 | Type II TA family toxin VapC |
| EFER_p54 | 230 | Rv | 51796 | 52026 | Type II TA family antitoxin AbrB |
| EFER_p55 | 350 | Fw | 52600 | 52950 | Hypothetical protein with helix-turn-helix domain |
| EFER_p56 | 743 | Fw | 53001 | 53744 | Hypothetical protein |
| EFER_p57 | 776 | Rv | 53741 | 54517 | Phage integrase |
| EFER_p58 | 290 | Rv | 54575 | 54865 | Hypothetical protein |

**Supplementary Table S2. Crystallographic data collection and refinement statistics.**

| | **BrxU Native 2.12 Å** | **BrxU Native 2.85 Å** | **BrxU Selenomethionine-derivatised 2.70 Å** |
|---|---|---|---|
| PDB ID Code | 7P9K | 7P9M | - |
| Number of crystals | 1 | 1 | 4 |
| Number of merged datasets | 2 | 5 | 15 |
| Crystallisation condition | 0.1 M Tris pH 7.5<br>0.2 M $(NH_4)_2SO_4$<br>20% PEG 3350 (w/v) | 0.1 M Tris pH 7.5<br>0.2 M $(NH_4)_2SO_4$<br>20% PEG 3350 (w/v) | 0.1 M Tris pH 7.5<br>0.2 M $(NH_4)_2SO_4$<br>20% PEG 3350 (w/v) |
| Beamline | DLS I24 | DLS I04 | DLS I24 |
| Wavelength, Å | 0.9781 | 0.9795 | 0.9786 |
| Resolution range, Å | 72.89 – 2.12 (2.16 – 2.12)* | 98.14 – 2.85 (2.96 – 2.85) | 128.93 – 2.70 (2.80 – 2.70) |
| Space group | C 1 2 1 | C 1 2 1 | C 1 2 1 |
| Unit cell, *a b c* (Å), α β γ (°) | 214.62 67.26 126.55, 90 102.55 90 | 196.88 68.37 129.59, 90 94.45 90 | 197.79 68.34 129.17, 90 95.05 90 |
| Total reflections | 191929 (9440) | 78775 (8839) | 4685930 (430888) |
| Unique reflections | 99627 (4879) | 40622 (4514) | 47486 (4406) |
| Multiplicity | 1.9 | 1.9 | 98.7 |
| Completeness (%) | 99.3 (98.6) | 99.8 (98.5) | 99.5 (95.2) |
| Mean I/sigma(I) | 5.7 | 9.8 | 14.5 |
| $R_{merge}$ | 0.066 (0.899) | 0.044 (1.021) | 0.492 (8.531) |
| $R_{meas}$ | 0.094 (1.271) | 0.063 (1.444) | 0.495 (8.575) |
| $CC_{1/2}$ | 0.994 (0.341) | 0.999 (0.368) | 0.995 (0.813) |
| $R_{work}$ | 0.201 | 0.266 | - |
| $R_{free}$ | 0.248 | 0.278 | - |
| No. of non-hydrogen atoms | 9848 | 9152 | - |
| Macromolecules | 9464 | 9136 | - |
| Ligands | 34 | 16 | - |
| Solvent | 350 | 0 | - |
| Protein Residues | 1160 | 1120 | - |
| RMSD (bonds, Å) | 0.008 | 0.013 | - |
| RMSD (angles, °) | 0.90 | 1.89 | - |
| Ramachandran favoured (%) | 96.44 | 94.72 | - |
| Ramachandran allowed (%) | 3.56 | 5.28 | - |
| Ramachandran outliers (%) | 0 | 0 | - |
| Average B-factor | 55.55 | 97.24 | - |

*Statistics for the highest-resolution shell are shown in parentheses.

**Supplementary Table S3. Oligonucleotides and plasmids used in this study.**

| Primer | Sequence | Notes |
|---|---|---|
| **pBAD30 cloning** | | |
| PF138 | CACACTTTGCTATGCCATAG | FWD pBAD30 MCS for sequencing |
| PF139 | GCTACTGCCGCCAGG | REV pBAD30 MCS for sequencing |
| TRB865 | TTTGAATTCAGGAGATATCTTATGCACCATCACCATCACCATGGAATGTATCAAGCGGGTGGAACA | FWD EcoRI, RBS, His$_6$, pEFER *brxU* |
| TRB866 | TTTTCTAGATTATTATTCTGTCTCTACGCTGCTTGC | REV XbaI pEFER *brxU* |
| **pSAT1-LIC cloning** | | |
| TRB851 | CAACAGCAGACGGGAGGTATGTATCAAGCGGGTGGAACA | FWD LIC, pEFER *brxU* |
| TRB852 | GCGAGAACCAAGGAAAGGTTATTATTCTGTCTCTACGCTGCTTGC | REV LIC, pEFER *brxU* |
| TRB873 | TTAATGCAGCTGATTAATACG | FWD pSAT1-LIC sequencing |
| TRB874 | AATCAATGAAACAGACACACC | REV pSAT1-LIC sequencing |
| TRB875 | TACTCAAGCTTATGCATGC | FWD pSAT1-LIC hSUMO2 sequencing |
| **Golden Gate Assembly and sequencing** | | |
| TRB740 | CTGGAATGTGCTCGTCTTCA | REV pEFER 700 sequencing |
| TRB741 | GCTTCCGATAAGATCGCCTA | REV pEFER 1400 sequencing |
| TRB742 | AGCAGTTCATACTTGGCTGGA | REV pEFER 2100 sequencing |
| TRB743 | CCAAAGAAGCCAGAAATCCA | REV pEFER 2800 sequencing |
| TRB744 | GCATCAATGTCTGCCTGAGA | REV pEFER 3500 sequencing |
| TRB745 | AATCAGCAATTGGCGTTCA | REV pEFER 4200 sequencing |
| TRB746 | GAAGATCCCGTCCAATCTGA | REV pEFER 4900 sequencing |
| TRB747 | CACTGTCTTTTTCTAACGCAGG | REV pEFER 5600 sequencing |
| TRB748 | GATCTTATTGTTCCACCCGC | REV pEFER 6300 sequencing |
| TRB749 | TAGGACAAAGTAGTGCCGCC | REV pEFER 7000 sequencing |
| TRB750 | CTTTGGGGTAAATGTGGTCC | REV pEFER 7700 sequencing |
| TRB751 | GCCTTTAGGGTTGTCTGGGT | REV pEFER 8400 sequencing |
| TRB752 | CTATGTCCAGGCCGTAGAGG | REV pEFER 9100 sequencing |
| TRB753 | AACTGGATTAGCGTTGAGATAA | REV pEFER 9800 sequencing |
| TRB754 | CTTTTGAGTTCAATATTGCTGC | REV pEFER 10500 sequencing |
| TRB755 | TGAGACATAGTGATTCGGCG | REV pEFER 11200 sequencing |
| TRB756 | TTGCCCATATTGAGGATGGT | REV pEFER 11900 sequencing |
| TRB757 | CAACTGTTGGCTAAGCACCTT | REV pEFER 12600 sequencing |
| TRB758 | GTATAACTCGGCAAAACGCC | REV pEFER 13300 sequencing |

| | | |
|---|---|---|
| TRB759 | CCGGTTGTTTCTCATGTTTTG | REV pEFER 14000 sequencing |
| TRB760 | GCTTAACGCTTACTTTGTCGATG | REV pEFER 14700 sequencing |
| TRB761 | TCACCAGTGTCTCAACGCTT | REV pEFER 15400 sequencing |
| TRB762 | GATCAGAAAACTTCGCCGAC | REV pEFER 16100 sequencing |
| TRB763 | CACTTCACATCATCGCCATC | REV pEFER 16800 sequencing |
| TRB1146 | TTTGGATCCGGTCTCGGGAGGTT ATGGCTGGATCACAGC | FW GGA BamHI BsaI Fragment 1 |
| TRB1147 | TTTCTGCAGGGTCTCGACCTCAA TCAAATCTTCCCG | REV GGA PstI BsaI Fragment 1 |
| TRB1148 | TTTGGATCCGGTCTCGAGGTCAT GGAGTGCACAATATGAAGG | FW GGA BamHI BsaI Fragment 2 |
| TRB1149 | TTTCTGCAGGGTCTCGCTGACA TTTCAACGTCAGTG | REV GGA PstI BsaI Fragment 2 |
| TRB1151 | TTTCTGCAGGGTCTCCGTTTCT TCAATGCGCTGTCTGC | REV GGA PstI BsaI Fragment 3 |
| TRB1152 | TTTGGATCCGGTCTCGAAACC TCTTCAATACCACAAATTATC | FW GGA BamHI BsaI Fragment 4 |
| TRB1153 | TTTCTGCAGGGTCTCGTGTCA CGAGAGCGGAAAC | REV GGA PstI BsaI Fragment 4 |
| TRB1154 | TTTGGATCCGGTCTCGGACAT TCCAAAGATGATTCTGGAGAA | FW GGA BamHI BsaI Fragment 5 |
| TRB1155 | TTTCTGCAGGGTCTCGCAAGA TCAAACTTTCCTAGCTG | REV GGA PstI BsaI Fragment 5 |
| TRB1156 | TTTGGATCCGGTCTCCCTTGAGA CGTCACTCTGGGCATATCTGAG | FW GGA BamHI BsaI Fragment 6 |
| TRB1157 | TTTCTGCAGGGTCTCCTAGAAAA AATCATCTTGGAATGCCAAATC | REV GGA PstI BsaI Fragment 6 |
| TRB1158 | TTTGGATCCGGTCTCCTCTAAGG CGCTGAGGTGA | FW GGA BamHI BsaI Fragment 7 |
| TRB1159 | TTTCTGCAGGGTCTCCATGGAGG ACAGAAACTGTCTACCG | REV GGA PstI BsaI Fragment 7 |
| TRB1160 | TTTCTGCAGGGTCTCCATGGCAA TCAAATCTTCCCG | REV GGA PstI BsaI Fragment 1 Control |
| TRB1161 | CTGCAGGAAGGTTTAAACGCAT TTAGG | FWD pGGA sequencing |
| TRB1162 | TAATACGACTCACTATAGGGAG ACGC | REV pGGA sequencing |
| TRB1203 | TTTCTGCAGGGTCTCGGCAATG AACTCACGTTCCGGTAACTG | REV GGA PstI BsaI Fragment 3 |
| TRB1211 | GCTGCAATGATACCGCGTGACCC ACGCTCACCGG | FWD QuikChange to remove BsaI from pUC19 |
| TRB1212 | CCGGTGAGCGTGGGTCACGCG GTATCATTGCAGC | REV QuikChange to remove BsaI from pUC19 |
| TRB1307 | TAATAACCGGAACGTGAGTTC | FWD Fragment 3 *ΔbrxU* |
| TRB1308 | TTACTTAATTCTTACTCGGTCG | REV Fragment 3 *ΔbrxU* |

| | | |
|---|---|---|
| TRB1309 | TAATAAGGTTTCCGCTCTCG | FWD Fragment 4 *ΔpglX* |
| TRB1310 | TTATTCTGTCTCTACGCT | REV Fragment 4 *ΔpglX* |
| TRB1358 | TTTGGATCCGGTCTCGGGAGGCC GCTTACGAGCGTGTA | FWD GGA BamHI BsaI Fragment 0 |
| TRB1359 | TTTCTGCAGGGTCTCGGTTTGGTT AGACGCCTCGGTAGT | REV GGA BamHI PstI Fragment 0 |

**Substrate amplification**

| | | |
|---|---|---|
| TRB1434 | ATTTTTAATTTAAAAGGAACTAGG TGAAGATCC | FWD pUC19 for generating DNA substrates with cytosine modifications |
| TRB1435 | TAATTTTTAAATCAATCTAAAGTAT ATATGAGTAA | REV pUC19 for generating DNA substrates with cytosine modifications |

| **Plasmid** | **Notes** | **Primers used** | **Reference** |
|---|---|---|---|
| pEFER | Ap$^R$,Tc$^R$,Sm$^R$ | - | ATCC 35469 |
| pEFER-*brxA*::Tn*5* | pEFER::Tn*5 ΔbrxA.* Ap$^R$,Tc$^R$,Sm$^R$,Km$^R$ | - | This study |
| pEFER-*str*::Tn*5* | pEFER::Tn*5 Δstr*. Ap$^R$,Tc$^R$,Km$^R$ | - | This study |
| pSAT1-LIC | Ap$^R$, IPTG-inducible | - | (42) |
| pTRB446 | pSAT1-LIC-*brxU*$^+$. Ap$^R$ | TRB851/852 | This study |
| pTRB479 | pUC19 derivative, mutated to remove BsaI sites, to allow cloning of GGA fragments. Ap$^R$ | TRB1211/1212 | This study |
| pTRB519 | pBAD30-*his*$_6$-*brxU*$^+$. Ap$^R$ | TRB865/866 | This study |
| pTRB563 | pBrxXL GGA assembly, fragments 0-7. Cm$^R$ | | This study |
| pTRB564 | pBrxXL-*ΔpglX* GGA assembly, fragments 0-7. Cm$^R$ | TRB1309/10 | This study |
| pTRB565 | pBrxXL-*ΔbrxU* GGA assembly, fragments 0-7. Cm$^R$ | TRB1307/8 | This study |
| pTRB566 | pBrxXL-*ΔbrxUΔpglX* GGA assembly, fragments 0-7. Cm$^R$ | TRB1307/8 TRB1309/10 | This study |
| pTRB603 | pBAD30-*his*$_6$-*brxU*$^+$ D474A. Ap$^R$ | Genscript synthesis | This study |
| pTRB604 | pBAD30-*his*$_6$-*brxU*$^+$ E528A. Ap$^R$ | Genscript synthesis | This study |
| pTRB605 | pBAD30-*his*$_6$-*brxU*$^+$ H475A. Ap$^R$ | Genscript synthesis | This study |
| pTRB606 | pBAD30-*his*$_6$-*brxU*$^+$ N485A. Ap$^R$ | Genscript synthesis | This study |
| pTRB607 | pBAD30-*his*$_6$-*brxU*$^+$ N519A. Ap$^R$ | Genscript synthesis | This study |
| pTRB608 | pBAD30-*his*$_6$-*brxU*$^+$ Q35A. Ap$^R$ | Genscript synthesis | This study |
| pTRB609 | pBAD30-*his*$_6$-*brxU*$^+$ Q101A. Ap$^R$ | Genscript synthesis | This study |
| pTRB610 | pBAD30-*his*$_6$-*brxU*$^+$ R38A. Ap$^R$ | Genscript synthesis | This study |
| pTRB611 | pBAD30-*his*$_6$-*brxU*$^+$ R102A. Ap$^R$ | Genscript synthesis | This study |
| pTRB612 | pBAD30-*his*$_6$-*brxU*$^+$ S42A. Ap$^R$ | Genscript synthesis | This study |
| pTRB613 | pBAD30-*his*$_6$-*brxU*$^+$ S42D. Ap$^R$ | Genscript synthesis | This study |

| | | | |
|---|---|---|---|
| pTRB614 | pBAD30-*his*$_6$-*brxU*$^+$ S42D/R38A double mutant. Ap$^R$ | Genscript synthesis | This study |
| pUC19 | Ap$^R$ | - | New England Biolabs |
